# Antibiotic prophylaxis and the gastrointestinal resistome in paediatric patients with acute lymphoblastic leukaemia: a cohort study with metagenomic sequencing analysis

**DOI:** 10.1016/s2666-5247(20)30202-0

**Published:** 2021-02-15

**Authors:** Elisa B Margolis, Hana Hakim, Ronald H Dallas, Kim J Allison, Jose Ferrolino, Yilun Sun, Ching-Hon Pui, Jiangwei Yao, Ti-Cheng Chang, Randall T Hayden, Sima Jeha, Elaine I Tuomanen, Li Tang, Jason W Rosch, Joshua Wolf

**Affiliations:** Department of Infectious Diseases, St Jude Children’s Research Hospital, Memphis, TN, USA; Department of Pediatrics, University of Tennessee Health Science Center, Memphis, TN, USA; Department of Infectious Diseases, St Jude Children’s Research Hospital, Memphis, TN, USA; Department of Pediatrics, University of Tennessee Health Science Center, Memphis, TN, USA; Department of Infectious Diseases, St Jude Children’s Research Hospital, Memphis, TN, USA; Department of Infectious Diseases, St Jude Children’s Research Hospital, Memphis, TN, USA; Department of Infectious Diseases, St Jude Children’s Research Hospital, Memphis, TN, USA; Department of Biostatistics, St Jude Children’s Research Hospital, Memphis, TN, USA; Department of Oncology, St Jude Children’s Research Hospital, Memphis, TN, USA; Department of Infectious Diseases, St Jude Children’s Research Hospital, Memphis, TN, USA; General Dynamics Information Technology, Falls Church, VA, USA; Department of Computational Biology, St Jude Children’s Research Hospital, Memphis, TN, USA; Department of Pathology, St Jude Children’s Research Hospital, Memphis, TN, USA; Department of Oncology, St Jude Children’s Research Hospital, Memphis, TN, USA; Department of Infectious Diseases, St Jude Children’s Research Hospital, Memphis, TN, USA; Department of Biostatistics, St Jude Children’s Research Hospital, Memphis, TN, USA; Department of Infectious Diseases, St Jude Children’s Research Hospital, Memphis, TN, USA; Department of Infectious Diseases, St Jude Children’s Research Hospital, Memphis, TN, USA; Department of Pediatrics, University of Tennessee Health Science Center, Memphis, TN, USA

## Abstract

**Background:**

Although antibiotic prophylaxis with levofloxacin can reduce the risk of serious infection in immunocompromised patients, the potential contribution of prophylaxis to antibiotic resistance is a major drawback. We aimed to identify the effects of levofloxacin prophylaxis, given to paediatric patients with acute lymphoblastic leukaemia to prevent infections during induction chemotherapy, on antibiotic resistance in gastrointestinal microbiota after completion of induction and consolidation therapy.

**Methods:**

This prospective, single-centre (St Jude Children’s Research Hospital, Memphis, TN, USA) cohort study included children (≤18 years) receiving therapy for newly diagnosed acute lymphoblastic leukaemia and who received either primary levofloxacin prophylaxis or no antibacterial prophylaxis (aside from *Pneumocystis jirovecii* prophylaxis with trimethoprim-sulfamethoxazole) and provided at least two stool samples, including one after completion of induction therapy. We used metagenomic sequencing to identify bacterial genes that confer resistance to fluoroquinolones, trimethoprim-sulfamethoxazole, or other antibiotics, and to identify point mutations in bacterial topoisomerases (*gyrA, parC*) that confer resistance to fluoroquinolones. We then used generalised linear mixed models to compare the prevalence and relative abundance of antibiotic resistance gene groups after completion of induction and consolidation therapy between participants who had received levofloxacin and those who received no prophylaxis.

**Findings:**

Between Feb 1, 2012, and April 30, 2016, 118 stool samples (32 baseline, 49 after induction, and 37 after consolidation) were collected from 49 evaluable participants; of these participants, 31 (63%) received levofloxacin prophylaxis during induction therapy and 18 (37%) received no antibacterial prophylaxis. Over the course of induction therapy, there was an overall increase in the relative abundance of trimethoprim-sulfamethoxazole resistance genes (estimated mean fold change 5·9, 95% CI 3·6–9·6; p<0·0001), which was not modified by levofloxacin prophylaxis (p=0·46). By contrast, the prevalence of topoisomerase point mutations increased over the course of induction therapy in levofloxacin recipients (mean prevalence 10·4% [95% CI 3·2–25·4] after induction therapy *vs* 3·7% [0·2–22·5] at baseline) but not other participants (0% *vs* 0%; p<0·0001). There was no significant difference between prophylaxis groups with respect to changes in aminoglycoside, β-lactam, vancomycin, or multidrug resistance genes after completion of induction or consolidation therapy.

**Interpretation:**

Analysing the gastrointestinal resistome can provide insights into the effects of antibiotics on the risk of antibiotic-resistant infections. In this study, antibiotic prophylaxis with trimethoprim-sulfamethoxazole or levofloxacin during induction therapy for acute lymphoblastic leukaemia appeared to increase the short-term and medium-term risk of colonisation with bacteria resistant to these antibiotics, but not to other drugs. More research is needed to determine the longer-term effects of antibacterial prophylaxis on colonisation with antibiotic-resistant bacteria.

**Funding:**

Children’s Infection Defense Center at St Jude Children’s Research Hospital, American Lebanese Syrian Associated Charities, and National Institutes of Health.

## Introduction

Although the overall survival for children with acute lymphoblastic leukaemia is more than 90%,^1^ infections remain a frequent cause of morbidity and mortality.^2–4^ Antimicrobial prophylaxis can help prevent these serious infections and reduce the risk of febrile neutropenia.^4–8^ Prophylaxis with levofloxacin, a broad-spectrum fluoroquinolone, has been shown to reduce the risk of serious bacterial infections and of *Clostridioides difficile* infection.^4–6^ Despite short-term efficacy,^4–6,8^ there is considerable concern that fluoroquinolone prophylaxis increases antibiotic resistance by selecting for antibiotic-resistant organisms in the gastrointestinal microbiome.^9^ This concern is supported by the fact that breakthrough infections during prophylaxis are typically resistant to the specific antibiotics used.^4,10–12^ An additional concern is that prophylaxis might also select for genes for resistance to other antibiotic classes, because resistance genes might be transmitted together on plasmids or co-exist in highly resistant organisms.^10–14^ However, an increase in resistance is not inevitable, because fluoroquinolone prophylaxis might also reduce the use of other broad-spectrum antibiotics for treating fever and infections.^4,15^

The collection of antibiotic resistance genes (ARGs) in the gut flora, the so-called gastrointestinal resistome, serves as a source of antibiotic resistance for bacteria and has been used to determine the potential for resistant infections in high-risk hosts.^16,17^ In this study, we aimed to investigate the gastrointestinal resistome using metagenomic sequencing of bacterial DNA from stool to determine whether antibiotic prophylaxis increased the prevalence or relative abundance of ARGs. The central hypothesis was that antibacterial prophylaxis would increase the burden of genes that confer resistance to the specific antibiotic given for prophylaxis and cross-resistance to other antibiotics.

## Methods

### Study design and participants

Patients with newly diagnosed acute lymphoblastic leukaemia treated at St Jude Children’s Research Hospital (Memphis, TN, USA), a quaternary paediatric cancer centre, in the Total Therapy XVI study (NCT00549848) between Jan 6, 2012, and Dec 1, 2015, and younger than 19 years were invited to participate in a prospective study of the gastrointestinal microbiome.^1,4,18^ The chemotherapy regimen comprised a 6-week induction phase, 8-week consolidation phase, and 120-week maintenance phase (appendix p 2). This substudy was restricted to patients who received either primary levofloxacin (levofloxacin prophylaxis group) or no antibacterial prophylaxis (aside from trimethoprim-sulfamethoxazole for *Pneumocystis jirovecii* prophylaxis; no prophylaxis group) and provided at least two stool samples, including one after completion of induction therapy. Detailed eligibility criteria are provided in the appendix (p 2).

The study was approved by the St Jude Institutional Review Board and written informed consent was obtained from each participant or their legally authorised representative, and assent was obtained from participants aged 7–18 years, before study procedures.

### Procedures

Faecal samples were collected prospectively for research purposes at predefined timepoints: during the first 3 days of induction therapy (baseline), after completion of induction therapy, and after completion of consolidation therapy (appendix pp 2, 19).^18^ Reasons for sample unavailability were not recorded. Demographic and leukaemia data were collected prospectively by the study team, and data on prescription of antibiotics for prophylaxis or treatment during the induction phase were collected retrospectively from electronic medical records.^4^ Antibiotic activity spectra were classified according to the Antibiotic Spectrum Index matrix.^19^

Participants routinely received prophylaxis against *P jirovecii* pneumonia with trimethoprim-sulfamethoxazole on 3 consecutive days per week, starting on week 3 of induction,^7^ and some additionally received primary antibacterial prophylaxis with levofloxacin. Between Feb 1, 2012, and Aug 12, 2014, primary levofloxacin prophylaxis was prescribed only at the clinician’s discretion and after Aug 13, 2014, it was routinely prescribed during periods of prolonged neutropenia.^4^ Other antibiotics were administered as clinically indicated. Antibiotic exposure was calculated as the number of days on which one or more systemic antibiotic was administered (excluding *P jirovecii* pneumonia prophylaxis), and the exposure to specific antibiotics or classes was calculated as the number of days each was administered. The cumulative antibiotic exposure was calculated as the sum of all specific antibiotic days to allow for a potential additive effect of co-administered antibiotics.^4^

Stool samples were frozen at −80°C to await DNA extraction as described in the appendix (p 3). Metagenomic sequencing was done using an Illumina HiSeq 2000 platform to generate 100-bp paired-end reads (appendix p 3). Sequence reads were trimmed and filtered for quality and those classified as human were removed with bbmap, using 95% identity and three maxindel reads. Non-host reads were aligned to the Comprehensive Antibiotic Resistance Database 2017, by use of bbmap (version 37.80) with semiperfect mode default settings, assigned to ARG sequences (genes associated with decreased susceptibility to an antibiotic), and retained if more than 50% of the gene had coverage.^20^ To determine whether substitutions, insertions, or deletions were present in the topoisomerase genes *gyrA* and *parC*, reads were aligned to a database of curated and well characterised mutations (appendix pp 5–9), and non-synonymous substitutions associated with fluoroquinolone resistance were identified. The composition and total bacterial reads were determined using Kraken taxonomic classification tools (appendix p 3). Sequences are available on the Sequence Read Archive, PRJNA656820.

Each ARG was classified on the basis of its mechanism and class as described in the Comprehensive Antibiotic Resistance Database 2017. β-lactamase genes were further subclassified according to different mechanisms (eg, serine β-lactamase or metallo-β-lactamase) and phylogenetic subclasses that had less than 60% sequence similarity between subclasses as described by Silveira and colleagues.^21^ Because of their high prevalence, there was a particular focus on serine β-lactamase classes SA1 (common in members of the Proteobacteria, Firmicutes, and Actinobacteria phyla) and SA2 (commonly found in members of the Bacteroidetes phylum).^21^

### Outcomes

The outcomes of interest were differences in the relative abundance or prevalence of specific bacterial ARGs between timepoints, or between prophylaxis groups (ie, no prophylaxis group *vs* levofloxacin prophylaxis group) at the same timepoint. The primary genes of interest were those that are known to confer resistance to specific antibiotics, including fluoro quinolones, trimethoprim-sulfamethoxazole, β-lactams, and vancomycin. To improve detection of changes over time or between prophylaxis groups, we used different approaches for analysis of common and rare resistance genes. For genes or groups of genes that were present in at least 50% of samples, relative abundance was the primary comparison between groups, whereas for less common genes, prevalence was the primary comparison. Relative abundance was defined as the number of reads assigned to specific ARG or class divided by the gene length and the total bacterial reads in the sample and is reported as reads per kilobase per million bacterial reads. Prevalence was defined as the proportion of samples containing the specific ARG or group of ARGs.

### Statistical analysis

A generalised linear mixed model (GLMM) was used to explore the association between levofloxacin prophylaxis and relative abundance or prevalence of specific bacterial antibiotic genes over time. Specifically, GLMMs were used to produce mean estimates of relative abundance or prevalence of each ARG or group of ARGs at each timepoint with 95% CIs to compare changes over time for each prophylaxis group and determine the significance of differences between prophylaxis groups. A negative binomial GLMM with a log link was used to estimate the fold change (the ratio of counts) in relative abundance between timepoints, and a binomial GLMM with a probit link with and without prophylaxis group effects was used to estimate prevalence of ARGs for each prophylaxis group and for the whole cohort at each timepoint. To describe the association between changes in resistome and microbiome composition, a linear model with or without random effects was applied to changes in the percentage relative abundance of a particular phylum and their association with the relative abundance of the ARG. Detailed statistical methods are described in the appendix (pp 3–4). Statistical analyses were done using SAS version 9.4 and R version 3.6.1. p values of less than 0·05 were considered statistically significant.

### Role of the funding source

The funder of the study had no role in study design, data collection, data analysis, data interpretation, or writing of the report.

## Results

245 patients were evaluated and 49 were included in the study (appendix p 20); 31 (63%) participants received levofloxacin prophylaxis and 18 (37%) received no primary antibacterial prophylaxis.^18^ Fecal samples were available at initial diagnosis for 32 (65%) participants, after induction for 49 (100%), and after consolidation for 37 (76%). Baseline characteristics of participants excluded for sample unavailability were similar to those included (appendix p 10), and sample availability was similar for participants who received levofloxacin or no prophylaxis (table 1). Demographic and leukaemia characteristics for participants who received levofloxacin prophylaxis were similar to those of participants who received no prophylaxis, but participants receiving levofloxacin prophylaxis had higher exposure to fluoroquinolones, lower exposure to most other antibiotics, and lower risk of febrile neutropenia and infections (table 1).^4^ In this cohort, there were 20 bloodstream infection episodes in 13 patients of which 15 had susceptibility testing available for analysis; there was no significant difference in susceptibility to levofloxacin or other antibiotics between participants who had received levofloxacin and those who received no primary prophylaxis (appendix p 10). Median non-host sequencing depth was 0·45 million reads (IQR 0·19 million–0·79 million) and did not differ significantly between timepoints or prophylaxis groups (appendix p 11). 157 discrete ARGs were detected (appendix p 11–14).

**Figure 1:**
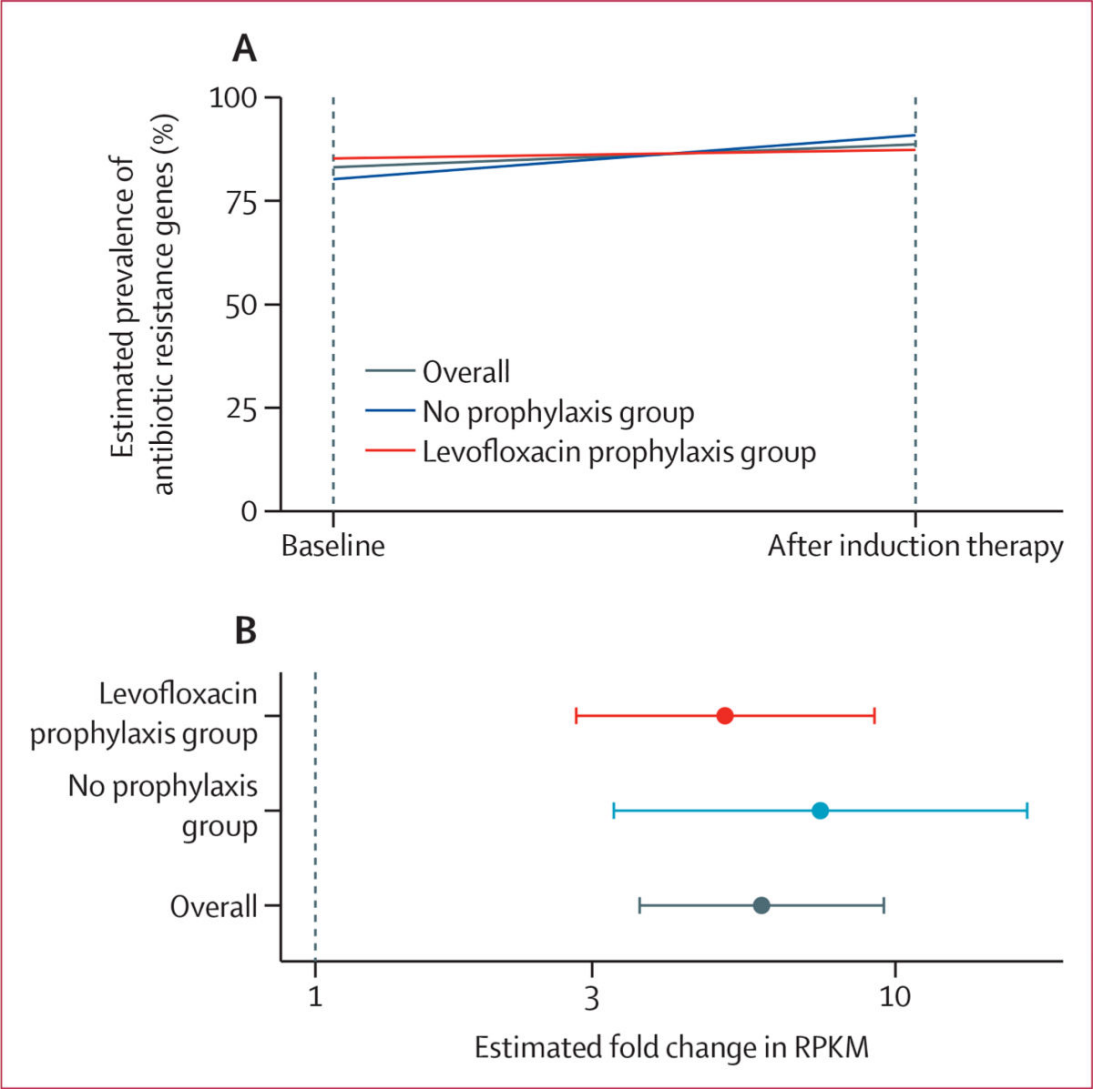
Changes in prevalence and relative abundance of trimethoprim-sulfamethoxazole antibiotic resistance genes during induction therapy for acute lymphoblastic leukaemia (A) Prevalence of trimethoprim-sulfamethoxazole resistance genes estimated from generalised linear mixed models, with and without prophylaxis group effect and accounting for sequence depth. The significance of the change in prevalence of trimethoprim-sulfamethoxazole resistance genes during induction therapy was not evaluated because the overall prevalence was too high. (B) Estimated changes in relative abundance of trimethoprim-sulfamethoxazole antibiotic resistance genes, estimated from generalised linear mixed models and expressed as the fold change in RPKM. RPKM=reads per kilobase gene length per million bacterial reads.

Almost all participants received prophylaxis against *P jirovecii* pneumonia with trimethoprim-sulfamethoxazole throughout therapy (table 1). Prevalence and relative abundance of genes conferring resistance to trimethoprim or sulfamethoxazole significantly increased during induction therapy (estimated mean fold change 5·9, 95% CI 3·6–9·6; p<0·0001; figure 1; appendix pp 15–16). This increase did not differ significantly between participants who received levofloxacin and those who received no prophylaxis (p=0·46).

**Figure 2:**
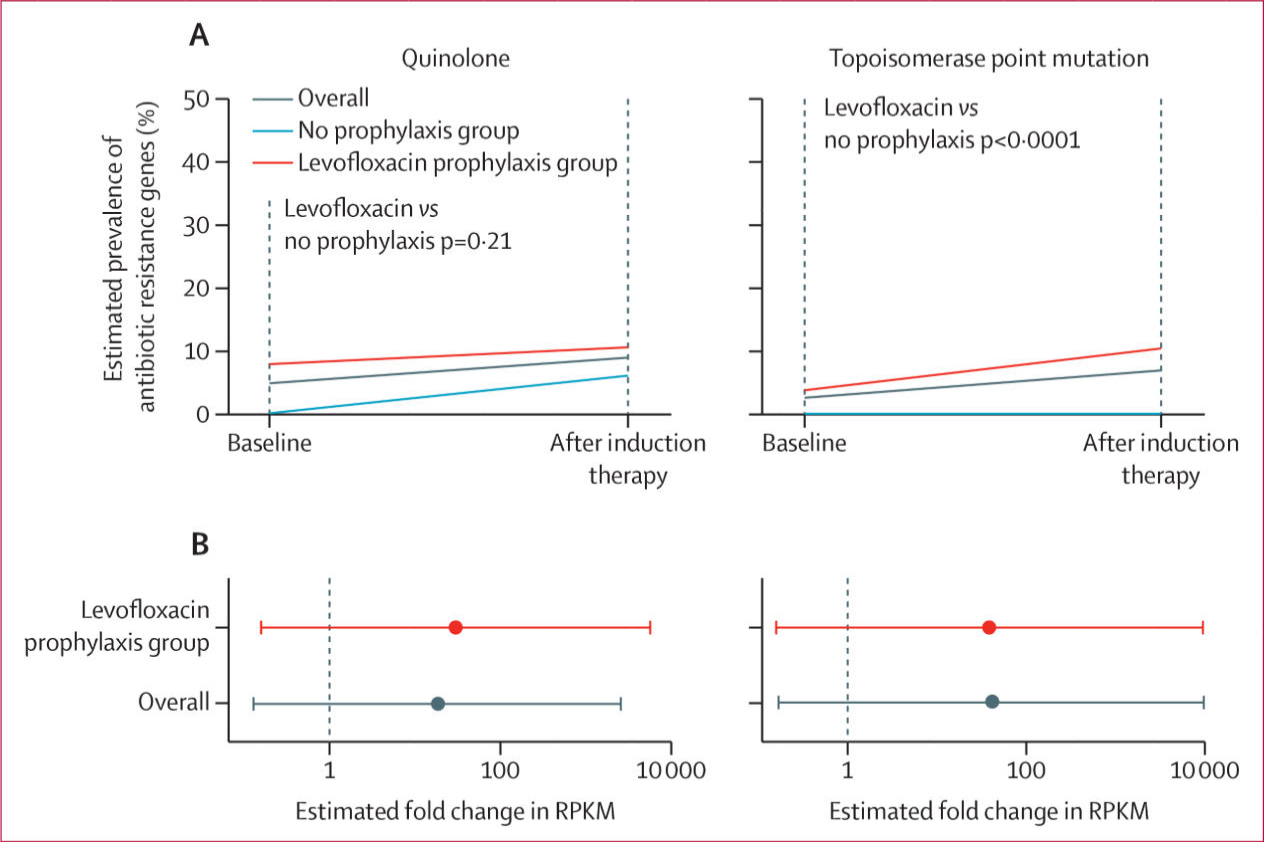
Changes in prevalence and relative abundance of fluoroquinolone antibiotic resistance genes and topoisomerase point mutations during induction therapy for acute lymphoblastic leukaemia (A) Prevalence of all quinolone antibiotic resistance genes and resistance mutations and of the topoisomerase point mutation subset (mutations in the *gyrA* and *parC*, which confer resistance to fluoroquinolones), estimated from generalised linear mixed models, with and without prophylaxis group effect and accounting for sequence depth. (B) Estimated changes in relative abundance of quinolone antibiotic resistance genes, primarily topoisomerase point mutations, estimated as the fold change in RPKM from generalised linear mixed models. RPKM=reads per kilobase gene length per million bacterial reads.

The prevalence of topoisomerase point mutations known to confer fluoroquinolone resistance increased during induction chemotherapy in participants receiving levofloxacin (10·4% [95% CI 3·2–25·4] after induction *vs* 3·7% [0·2–22·5] at baseline) but not those receiving no prophylaxis (0% *vs* 0%; p<0·0001; figure 2; appendix p 15). However, the estimated prevalence remained low, reaching a maximum of 10·4% after the completion of induction in participants who received levofloxacin, and increasing to 15·1% after the 8-week consolidation phase of chemotherapy, when the fluoroquinolone pressure had been removed. In contrast with topoisomerase point mutations, acquisition of specific fluoroquinolone resistance genes was too infrequent for any effect of prophylaxis to be detected (appendix p 16).

**Figure 3:**
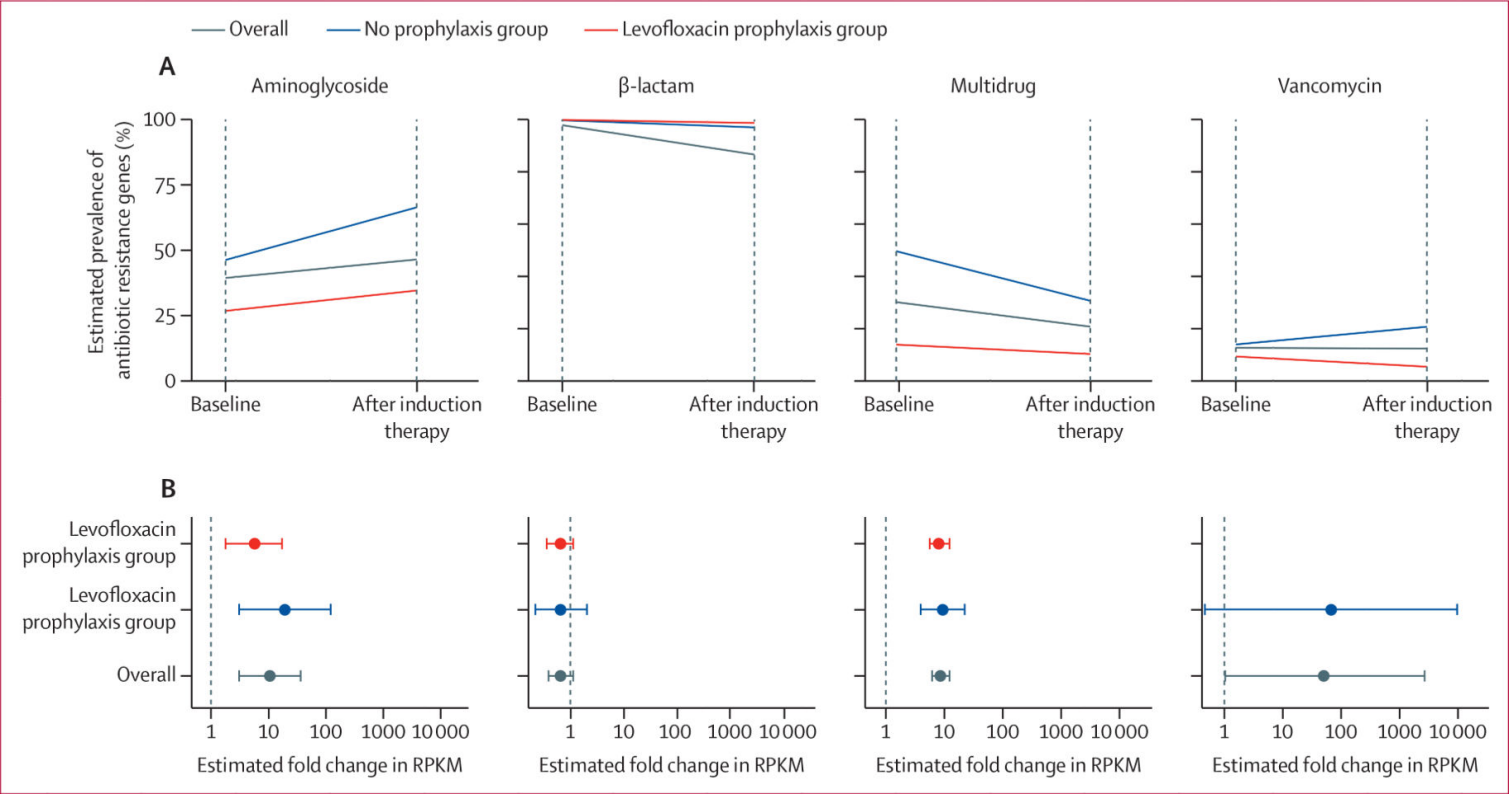
Changes in prevalence and relative abundance of selected antibiotic resistance gene classes during induction therapy for acute lymphoblastic leukaemia (A) Prevalence of aminoglycoside, β-lactam, multidrug, and vancomycin resistance genes estimated from generalised linear mixed models. (B) Estimated changes in relative abundance of aminoglycoside, β-lactam, multidrug, and vancomycin resistance genes estimated from generalised linear mixed models. RPKM=reads per kilobase per million bacterial reads.

During induction therapy, there was no significant difference in the prevalence, but a significant increase in the relative abundance, of aminoglycoside (mean fold change 10·5 [95% CI 3·2–34·8]) and multidrug resistance genes (mean fold change 8·6 [95% CI 5·8–12·6]), regardless of levofloxacin prophylaxis (appendix pp 15–16). There was no significant difference in the prevalence (12·3% [5·3–24·1] *vs* 12·5% [4·3–28·0]; p=0·97) or relative abundance (mean fold change 51·2 [1·0–2739·3]) of vancomycin resistance genes over the same period (figure 3; appendix pp 15–16). The SA1 group of β-lactamases increased in relative abundance (mean fold change 4·4 [0·7–26·8]) and prevalence (13·7% [6·4–25·2] *vs* 10·5% [3·0–26·4]; p=0·67) during induction chemotherapy, whereas the SA2 group was ubiquitous but decreased in relative abundance (mean fold change 0·6 [0·3–1·1]; appendix pp 15–16, 21).^21^

In addition to the effects of prophylaxis exposure, we also identified independent effects of changes in microbiome composition on the resistome (table 2; appendix p 17). For example, a decrease in the relative abundance of serine β-lactamase class A2 genes, which are typically found in members of the Bacteroidetes phylum, correlated with a decrease in the percentage relative abundance of those organisms (p=0·0014). In a multivariable model, the association between the relative abundance of SA2 genes and the relative abundance of Bacteroidetes phylum (p=0·014) was independent of exposure to antibiotics active against *Bacteroides fragilis* (p=0·60; table 2). There was also no association between changes in SA2 genes and exposure to anti-pseudomonal β-lactam antibiotics (p=0·72; appendix p 18).We evaluated the association between the change in relative abundance of ARG classes during induction therapy and other antibiotic exposure during the same period (appendix p 18). There was no association between cumulative antibiotic exposure and changes in the relative abundance of multidrug resistance genes (table 2). However, exposure to anti-pseudomonal β-lactam drugs (p=0·021) and an increase in members of the Proteobacteria phylum (p=0·0026) were each associated with an increased relative abundance of multidrug resistance genes, independent of prophylaxis group, but this association did not remain significant in the multivariable analysis (table 2). Similarly, the relative abundance of vancomycin resistance genes was associated with exposure to antibiotics active against *B fragilis* (p=0·0005), but not with a change in the percentage relative abundance of members of the Firmicutes phylum (p=0·99; table 2).

## Discussion

We used metagenomic sequencing to evaluate the effect of antibacterial prophylaxis on the resistome in the lower gastrointestinal tract, a reservoir of potentially pathogenic organisms. Use of fluoro quinolone prophylaxis reduces use of other antibiotics, so the overall effect on antibiotic resistance is unknown. Our approach enabled us to evaluate the effects of prophylaxis more sensitively than relying on rare events, such as breakthrough infection with antibiotic-resistant pathogens. We found a slight increase in the frequency of bacterial gene mutations that confer resistance to fluoroquinolones in participants receiving levofloxacin and an increase in genes that confer resistance to trimethoprim-sulfamethoxazole in participants receiving trimethoprim-sulfamethoxazole, which was not modified by levofloxacin exposure. By contrast, despite an a-priori hypothesis, there was no evidence that fluoroquinolone prophylaxis had any effect on cross-class resistance to any other antibiotics.^11,22,23^ These data show that resistome analysis based on metagenomic sequencing can be used to assess potential drawbacks of antibacterial prophylaxis in high-risk populations.

These findings also support the hypothesis that an increase in breakthrough infections due to organisms resistant to the prophylactic regimen is, at least in part, associated with selection for colonisation with these organisms.^4,10–12,14^ These results are similar to findings from another study^17^ that compared the effects of trimethoprim and fluoroquinolone prophylaxis on the resistome in adults with haematological malignancies. That study found a larger effect for trimethoprim-sulfamethoxazole than fluoroquinolone, but in our study, almost all participants received trimethoprim-sulfamethoxazole, so no comparison was possible. Furthermore, the effects of prophylaxis might differ between children and adults, because children might have distinct microbiome characteristics and a higher incidence of fever necessitating broad-spectrum antibiotic treatment.

With regard to long-term safety and efficacy, this study raises concerns of potential drawbacks of prolonged antibacterial prophylaxis for individual patients and for institutions, because prophylaxis could become less effective over time for individuals or institutions.^10,11,25^ Additionally, antibacterial prophylaxis provides an ideal scenario for the evolution of antibiotic resistance, because prolonged exposure to low concentrations of antibiotic might enable multiple lineages to sequentially acquire genes or mutations that each confer small resistance gains. However, although detectable, the increase in the prevalence of fluoroquinolone resistance genes was small; the proportion of participants harbouring topoisomerase point mutations remained small, and no large increase in the acquisition of other fluoroquinolone resistance genes occurred. This finding suggests that levofloxacin prophylaxis was not a very strong selective force in this population, which might be associated with the effect of levofloxacin being obscured by numerous other concomitant microbiome disruptors and selective forces, such as anticancer chemotherapy, immune dysfunction, damage to the gut epithelium, and dietary changes.^4^ The role of other microbiome disruptors is supported by the strong associations we found between selection for some resistance genes and microbiome composition or exposure to antibiotics for treating suspected infection. Other possible explanations are that paediatric patients have fewer fluoroquinolone resistance genes in their gastrointestinal microbiome than adults or that sampling was too far apart to capture dynamic changes attributable to levofloxacin prophylaxis. Further studies that examine other populations will be necessary to confirm whether selection by antibiotics differs in paediatric leukaemia resistomes and similar adult populations.^26^ Follow-up studies of individuals who receive antibacterial prophylaxis and surveillance in institutions that prescribe antibacterial prophylaxis might help elucidate the long-term consequences of such prophylaxis.

We observed an absence of an apparent effect of levofloxacin prophylaxis on the acquisition of cross-resistance to other antibiotics. Other clinical studies have suggested either an increase^11,12,25^ or no increase in breakthrough infections with cross-resistance to other antibiotics,^4–6,17^ but in our study, breakthrough infection was too rare to assess the effect.^4^ Therefore, analysing the lower gastrointestinal microbiome, a potential reservoir of resistant pathogens, is a sensitive way to address this issue.^27^ The absence of increased cross-resistance in the gastrointestinal reservoir in this study is consistent with data from laboratory experiments and animal models suggesting that fluoroquinolone prophylaxis actually protects against the development of crossresistance.^28,29^ This finding might be partly explained by a reduction in exposure to other antibiotics, such as β-lactams, cephalosporins, aminoglycosides, and glycopeptides, which might cause cross-resistance through either antibiotic selection pressure or changes in microbiome composition.

The gastrointestinal resistome provides a lens through which to view the larger reservoir from which drug-resistant organisms are likely to become clinically and epidemiologically significant, especially in a high-risk population. However, the examination of the resistome should be approached with caution. Not all resistance genes are sufficient to cause resistance alone; many could be under strong selection for non-resistance functions (eg, stringent stress response), and changes in their relative abundance could correspond to changes in the relative proportions of microbiome members with low virulence potential. Similarly, not all resistance genes confer resistance in all microbiomes. For example, although we noted that an abundance of the *tetX* gene was strongly associated with a relative abundance of Bacteroidetes, this gene confers tetracycline resistance only in aerobic bacteria, not in the anaerobic Bacteroidetes.^30^ Therefore, an increase in *tetX* in that context does not suggest an increase in resistance to tetracycline antibiotics. Furthermore, not all genes will be on plasmids, within organisms with transmission or recombination potential, or within genomes that allow compensatory evolution. Although this study was able to characterise the gut reservoir of resistance genes, future studies that integrate methods to investigate whether these genes are found in specific pathogens, on plasmids, or associated with insertion sequences,^17^ either through association^27^ or by clustering co-abundant genes,^31^ will be crucial to refining the prediction of infections and the spread of antibiotic-resistant organisms from this reservoir. In this study, we used two complementary metrics to evaluate the effect of prophylaxis exposure and changes in ARGs: prevalence and relative abundance. For most ARGs, both prevalence and relative abundance are reported, but in situations in which prevalence was very high, comparing the change in relative abundance provided a more sensitive measure of the potential effects of exposures.

The strengths of the study include the high-resolution clinical and laboratory data, the ability to account for specific antibiotic exposures, and the relatively large number of participants with diverse baseline resistomes. We reduced misclassification errors by directly extracting clinical and laboratory data, and we used statistical analysis tools that enabled us to control for the effects of several discrete characteristics and exposures and for potential interactions between these. Differences in resistomes between groups (eg, higher prevalence of aminoglycoside or lower prevalence of quinolone resistance genes at baseline in participants who received no prophylaxis) were accounted for at subsequent timepoints. We also enrolled a population of patients with documented heterogeneous baseline resistomes, which enabled us to control for the potential effect of the initial resistome at subsequent timepoints. Together, these strengths enabled us to specifically evaluate the potential effects of the prophylaxis regimen on the development of resistance in the lower gastrointestinal microbiome.

This study also had some limitations. Because development of cross-resistance might be a rare event, and because of the small sample size, we are unable to exclude it as a possibility entirely. Participants receiving quinolone prophylaxis were mostly treated after 2014, so temporal changes might have confounded the results. Resistomes characterised in this study might underestimate the extent of resistance within the gut microbiomes, because sequence depth can affect the detection of ARGs, and the average sample non-host read count of 0·45 million was shallow for comprehensive characterisation. However, this approach was practical and adequate for a comparison study of this magnitude, and sampling depth was similar between exposure groups. Furthermore, sensitivity might be affected by microbiome composition, because resistance genes might be unevenly characterised across or be intrinsic to different bacteria, and the lack of functional characterisation can lead to a high false-positive rate, because many gene functions have been inferred solely by homology^24^ and many are regulatory components of operons that could have functions that are not associated with antibiotic resistance. Additionally, resistance conferred by small mutations in existing genes, transcriptional modification, or gene duplication is not detected by this approach. We aimed to account for some of these issues by including a separate analysis for topoisomerase point mutations to maximise the detection of fluoroquinolone resistance, by normalising the reporting of the abundance of resistance genes for sequence depth, and by aggregating genes that confer resistance to each antibiotic target class. Owing to the very low number of quinolone resistance genes other than topoisomerase point mutations, the effect of levofloxacin prophylaxis on this group of ARGs was not evaluable in this study. Individual ARGs and mutations in topoisomerase point mutations can have differing effects on the minimum inhibitory concentration for bacteria depending on the genetic background and the presence of other genes or mutations. In this study, the gut microbial population was not cultivated, so the effect of ARGs on minimum inhibitory concentrations could not be directly measured. Therefore, these metagenomic analyses are intended to augment, rather than replace, phenotypic analyses by providing a snapshot of the antibiotic resistance reservoir, and they do not indicate whether a resistant organism will go on to cause invasive disease.

Overall, in this cohort of children and adolescents with acute lymphoblastic leukaemia, prophylaxis with trimethoprim-sulfamethoxazole or levofloxacin during chemotherapy was associated with an increase in the prevalence of resistance genes to these specific antibiotics, but not with cross-resistance to other antibiotics. Although the selective effect of levofloxacin appeared small, an increase in the frequency of fluoroquinolone resistance persisted for at least 2 months after exposure. By contrast, the short-term protection against serious infections provided by fluoroquinolone prophylaxis ^4,5^ did not come at the cost of selection for genes that confer cross-resistance to other antibiotics in the gastrointestinal reservoir.^5,6^ Further studies are needed to evaluate the clinical and epidemiological effects of long-term use of these drugs on the resistome and on breakthrough infections with resistant organisms, as well as to evaluate alternative strategies, such as probiotics and other methods for manipulation of the microbiome. Antimicrobial stewardship remains essential in this vulnerable population and should be considered in all decisions regarding antibacterial use.

## Supplementary Material

1

## Figures and Tables

**Table 1:** Characteristics of study participants

	No prophylaxis (N=18)	Levofloxacin (N=31)	p value
Age, years	7·3 (5·3)	8·8 (5)	0·33
Sex	··	··	0·38
Female	6 (33%)	15 (48%)	··
Male	12 (67%)	16 (52%)	··
Race (self-reported)	··	··	0·39
White	17 (94%)	26 (84%)	··
Black	1 (6%)	5 (16%)	··
Leukaemia type	··	··	>0·99
B cell	16 (89%)	27 (87%)	··
T cell	2 (11%)	4 (13%)	··
Leukaemia risk category	··	··	>0.99
Low	10 (56%)	16 (52%)	··
Standard	7 (39%)	13 (42%)	··
High	1 (6%)	2 (6%)	··
*Pneumocystis jirovecii* prophylaxis	··	··	>0·99
Trimethoprimsulfamethoxazole	17 (94·4%)	30 (96·8%)	··
Pentamidine only	1 (5·6%)	1 (3·2%)	··
Infection during induction			
Febrile neutropenia	12 (67%)	13 (42%)	0·14
Probable bacterial infection	7 (39%)	5 (16%)	0·094
Bloodstream infection	2 (11%)	2 (6%)	0·62
*Clostridioides difficile*	3 (17%)	0 (0%)	0·044
Antibiotic exposure, days			
Fluoroquinolone	4·7 (11·2)	29·3 (11)	<0·0001
Cefepime or ceftazidime	10·7 (10)	5 (5·4)	0·035
Meropenem	4·2 (6·3)	0·8 (2·1)	0·035
Vancomycin	6·7 (5·7)	6·3 (8)	0·85
Aminoglycoside	1·3 (2·8)	0·1 (0·2)	0·080
Anti-pseudomonal β-lactam	15·1 (13·5)	5·8 (5·7)	0·011
*Bacteroides fragilis*-active antibiotics	6·3 (7·7)	1·9 (4·3)	0·037
Cumulative antibiotic exposure*	31·7 (26·2)	43·8 (18)	0·094
Fecal samples available			
Baseline	13 (72%)	19 (61%)	0·54
After induction therapy	18 (100%)	31 (100%)	>0·99
After consolidation therapy	13 (72%)	24 (77%)	0·74

Data are mean (SD) or n (%). p value based on Student’s *t* test or Fisher’s exact test, as dictated by variable type.

* Each calendar day can contribute more than 1 day of exposure if more than one antibiotic is administered.

**Table 2:** Association between log_10_ fold changes in antimicrobial resistance gene relative abundance during induction chemotherapy and antibiotic exposure or microbiome compositional changes

	Adjusted for prophylaxis group		Adjusted for other covariates	
	Estimate (standard error)*	p value	Estimate (standard error)	p value
**Multidrug antimicrobial resistance genes**				
Cumulative antibiotic exposure	0·004 (0·013)	0·75	··	··
Anti-pseudomonal β-lactam exposure	0·062 (0·025)	0·021	0·025 (0·023)†	0·30
Change in Proteobacteria percentage relative abundance	0·028 (0·012)	0·026	0·023 (0·013)‡	0·087
**Quinolone resistance genes**				
Cumulative antibiotic exposure	−0·008 (0·016)	0·63	··	··
Fluoroquinolone exposure	0·002 (0·020)	0·93	··	··
Change in Proteobacteria percentage relative abundance	0·014 (0·016)	0·040	··	··
**SA2 resistance genes**				
Anti-pseudomonal β-lactam exposure	−0·010 (0·028)	0·72	··	··
*Bacteroides fragilis*-active antibiotic exposure	−0·070 (0·037)	0·069	−0·019 (0·035)§	0·60
Change in Bacteroidetes percentage relative abundance	0·014 (0·004)	0·0014	0·012 (0·004)¶	0·014
**Vancomycin resistance genes**				
Vancomycin exposure	0·045 (0·026)	0·093	··	··
*B fragilis*-active antibiotic exposure	0·106 (0·027)	0·0005	0·103 (0·026)ǁ	0·0004
Change in Firmicutes percentage relative abundance	0·000 (0·006)	0·99	−0·001 (0·005)**	0·77

Estimate represents estimated log_10_ fold change in the relative abundance of each resistance gene that is associated with a 1-day increase in antibiotic exposure or a 1% increase in percentage relative abundance of a specific group of bacteria.

* Estimates included adjustment for prophylaxis group.

† Estimate included adjustment for Proteobacteria percentage relative abundance.

‡ Estimate included adjustment for anti-pseudomonal β-lactam exposure.

§ Estimate included adjustment for Bacteroidetes percentage relative abundance.

¶ Estimate included adjustment for B fragilis-active antibiotic exposure.

ǁ Estimate included adjustment for Firmicutes percentage relative abundance.

** Estimate included adjustment for B fragilis-active antibiotic exposure.

## Research in context

### Evidence before this study

We searched PubMed for studies published in any language between database inception and April 4, 2020, with the term “(Fluoroquinolone* OR Levofloxacin OR Pefloxacin OR Norfloxacin OR Ofloxacin) AND (Cancer OR Leukemia) AND (Prophyla*) AND (Pediatric OR Child OR Children Or Infant*) NOT Review[PT]” to identify original studies that evaluated the effect of fluoroquinolone antibacterial prophylaxis on risk of antibacterial resistance in children with leukaemia. A 2019 systematic review of randomised trials that included both paediatric and adult patients reported a significant increase in infections resistant to fluoroquinolone antibiotics, but did not report an analysis limited to paediatric patients. We found four relevant parallel-group trials and six observational comparative studies; of these, three found an increase in resistant infections, five found no increase in resistance, and two did not report this outcome.

### Added value of this study

By use of metagenomic sequencing of bacterial DNA from stool samples collected before and after induction therapy in a cohort of paediatric patients with acute lymphoblastic leukaemia who did or did not receive levofloxacin prophylaxis, we were able to show that levofloxacin prophylaxis was associated with a slight increase in resistance to fluoroquinolone antibiotics, but not to other antibiotics. This new information will aid in the understanding of the overall effects of levofloxacin prophylaxis.

### Implications of all the available evidence

Fluoroquinolone prophylaxis reduces the risk of serious infections in neutropenic paediatric patients with leukaemia and does not substantially increase the short-term risk of antibiotic resistance in bacteria colonising the gastrointestinal tract. Long-term efficacy, safety, and resistome data are needed to further assist clinicians making decisions about use of antibiotics to prevent infections in immunocompromised patients.
