# Supplementary material for: Antibiotic prophylaxis and the gastrointestinal resistome in paediatric patients with acute lymphoblastic leukaemia: a cohort study with metagenomic sequencing analysis
Source: Lancet Microbe. Author manuscript; Available in PMC 2021 Aug 4. (PMC8336918; doi:10.1016/s2666-5247(20)30202-0)
Supplement: 1 [file NIHMS1689263-supplement-1.pdf]

THE LANCET
Microbe

Supplementary appendix

This appendix formed part of the original submission and has been peer reviewed. We post it as supplied by the authors.

Supplement to: Margolis EB, Hakim H, Dallas RH, et al. Antibiotic prophylaxis and the gastrointestinal resistome in paediatric patients with acute lymphoblastic leukaemia: a cohort study with metagenomic sequencing analysis. *Lancet Microbe* 2021; published online Feb 15. https://doi.org/10.1016/S2666-5247(20)30202-0.

Contents

1 Supplemental Methods **2**
1.1 Eligibility criteria for the Total Therapy XVI microbiome and resistome study 2
1.2 Risk adapted chemotherapy regimen and fecal sample collection 2
1.3 Levofloxacin prophylaxis 3
1.4 Trimethoprim-Sulfamethoxazole prophylaxis 3
1.5 Supplemental statistical methods 3

2 Supplemental Tables **5**
2.1 Dataset of characterized SNPs in topisomerase genes gyrA and parC associated with fluoroquinolone resistance 5
2.2 Comparison of characteristics of included with excluded participants 10
2.3 Antibiotic susceptibility patterns in isolates from bloodstream infection 10
2.4 Comparison of sequencing depth between prophylaxis groups 11
2.5 Detection frequency of ARGs among 118 samples 11
2.6 Estimated prevalence of antibiotic resistance gene classes at each time point based on GLMMs for binary outcomes 15
2.7 Estimated mean fold change of abundance of antibiotic resistance gene classes at each time point based on negative binomial GLMMs 16
2.8 Specific quinolone resistance antibiotic resistance genes 16
2.9 Association between ARG and microbiome composition 17
2.10 Association between antibiotic use and antibiotic resistance gene abundance 18

3 Supplemental Figures **19**
3.1 Study time course 19
3.2 Flow diagram summarizing eligibility and inclusion 20
3.3 Serine-β-lactamases class A prevalence and abundance 21

4 References **21**

1 Supplemental Methods

1.1 Eligibility criteria for the Total Therapy XVI microbiome and resistome study

Inclusion criteria:

1. Diagnosis of precursor B-cell or precursor T-cell ALL by immunophenotyping.
2. Age $\leq$18 years (inclusive).
3. Limited prior therapy, including systemic glucocorticoids for one week or less, one dose of vincristine, emergency radiation therapy to the mediastinum and one dose of intrathecal chemotherapy.
4. Written, informed consent and assent following Institutional Review Board, NCI, FDA, and OHRP Guidelines.
5. Evaluable fecal sample collected after completion of induction therapy.*
6. At least 1 evaluable fecal sample at baseline or after completion of consolidation therapy.*

Exclusion criteria:

1. Participants with prior therapy, other than therapy specified in inclusion criteria.
2. Participants who are pregnant or lactating.
3. Inability or unwillingness of research participant or legal guardian/representative to give written informed consent.
4. Received chemotherapy for longer than 72 hours.
5. Received antimicrobial agents for more than 72 hours in the preceding week.
6. Clinically or microbiologically proven diarrheal illness.
7. Receipt of antibacterial prophylaxis other than levofloxacin.*
8. Suspected or proven infection requiring antibiotic therapy within the first week of induction therapy or prior to onset of neutropenia (ANC $\leq$ 500 cells/mm3).*

Criteria specific to this resistome analysis are marked*. All other criteria were specific for Total Therapy XVI microbiome study [1, 2]

1.2 Risk adapted chemotherapy regimen and fecal sample collection

Fecal samples were collected without specific media by the participant or nursing staff and immediately refrigerated until aliquoted and frozen at -80° C awaiting sequencing. The baseline sample was collected at study enrollment within 72 hours of initiation of induction chemotherapy. The chemotherapy regimen began with a 42–day induction period comprising prednisone (40 mg/m^2/day for 28 days), vincristine (1.5 mg/m^2 weekly for 4 doses), daunorubicin (25 mg/m^2 weekly for two doses), and pegaspargase (3000 units/m^2 weekly for one or two doses), followed by cyclophosphamide(1000 mg/m^2 once or 300 mg/m^{2}for 4 doses), cytarabine(75 mg/m^2 for 8 doses), and tioguanine (60 mg/m^2 daily for 14 doses) plus risk-directed intrathecal chemotherapy. The post-induction fecal sample was collected after completion of this course of therapy. This was followed by an 8–week consolidation period comprising high dose methotrexate (2.5 g/m^2 or targeted by plasma levels every other week for 4 doses), mercaptopurine (50 mg/m^2 daily for 56 days), risk-directed intrathecal chemotherapy, plus pegaspargase for standard- and high-risk patients only. Following this participants entered a 120-week continuation therapy phase comprising mercaptopurine, dexamethasone and vincristine, plus risk-directed doxorubicin and pegaspargase. There were also two 3-week periods of more intensive chemotherapy referred to as Re-induction I (weeks 7-9) and Re-induction II (weeks 17-20) comprising pegaspargase, dexamethasone, vincristine and doxorubicin, plus risk-directed cytarabine. The post-consolidation fecal sample was collected 6 weeks after completion of consolidation, prior to the Re-induction I phase, when all participants returned to the hospital. [2]

1.3 Levofloxacin prophylaxis

Participants who received levofloxacin prophylaxis were prescribed oral or IV levofloxacin during periods of neutropenia (ANC $\leq$ 500 cells/mm^3) expected to last at least 7 days, in the opinion of the treating clinician. Dosing for levofloxacin prophylaxis was as follows: age $<$ 5 years, 10 mg/kg IV or PO every 12 h (max. 250 mg every 12 h); age $\geq$ 5 years, 10 mg/kg IV or PO daily (max. 500mg daily).[3]

1.4 Trimethoprim-Sulfamethoxazole prophylaxis

Participants who received trimethoprim-sulfamethoxazole prophylaxis were prescribed oral trimethoprim-sulfamethoxazole starting on week 3 of induction. Dosing was 75 mg/m^2 trimethoprim plus 375 mg/m^2 sulfamethoxazole (Maximum 160/800 mg per dose), twice daily on 3 consecutive days per week.

Supplemental metagenomic sequencing methods

Stool samples frozen at -80° C were diluted to 10% w/v with nuclease-free PBS (Lonza, Wakersfield, MD, USA) and 20 mg frozen stool equivalent was extracted (FastDNA Kit, MP Biomedicals, Santa Ana, CA, USA) using bead-beater (FastPrep, MP Biomedicals, Santa Ana, CA, USA).

All library preparations and sequencing steps were performed by the Hartwell Center at St. Jude Children's Research Hospital. Illumina TruSeq libraries were prepared according to the manufacturer's instructions (Illumina, San Diego, CA, USA) and run on an Illumina HiSeq 2000 platform with Nextera XT Index Kit (Illumina, San Diego, CA, USA) and Hiseq reagent kit (Illumina, San Diego, CA, USA). Reads were trimmed for quality when the average phred score across nucleotides fell below 10 using AfterQC (v 0.9.0). Nextera Transposase adapters were removed allowing a maximum of one mismatch. Reads classified as human were removed with bbmap, using 95% identity and three maxindel reads. Non-host reads were then aligned to the Comprehensive Antibiotic Resistance Database 2017 (CARD; downloaded August 22, 2018) by using bbmap (v 37.80) with semiperfect mode default settings. The non-host read sequencing depth varied from 43 thousand to 6 million reads, with a median of 0.45 million reads. Reads were assigned to antibiotic resistance gene (ARG) sequences and retained only if $>$ 50% of the gene had coverage[4].

To determine whether substitutions, insertions, and/or deletions were present in the topoisomerase genes *gyrA* and *parC*, reads were aligned with BWA alignment to a database of curated and well-characterized mutations associated with fluoroquinolone resistance (Appendix pp 3–8); SNPfinder (release March 27, 2017) was used to identify nonsynonymous substitutions associated with fluoroquinolone resistance. The composition and total bacterial reads were determined using Kraken taxonomic classification tools (Kraken-filter script), with a threshold of 0.05 against a database containing the complete genomes in RefSeq for bacteria, viruses, fungi, protozoa, archaea, and humans[5]. Sequences are available on SRA PRJNA656820.

Each ARG was classified based on its mechanism and class as described in CARD. β-Lactamase genes were further subclassified according to different mechanisms (e.g. serine or metallo) and phylogenetic subclasses that had less than 60% sequence similarity between subclasses as described by Chaves Silveira and colleagues.[6] Because of their high prevalence, there was a particular focus on serine β-lactamase classes SA1 (common in members of the Proteobacteria, Firmicutes, and Actinobacteria phyla) and SA2 (commonly found in members of the Bacteroidetes phylum).[6]

1.5 Supplemental statistical methods

Student's t- and Fisher's exact tests were used to evaluate the significance of differences in participant characteristics and in proportions of antibiotic-resistant breakthrough infections between groups. Student's t-test was used for continuous variables and Fisher's exact test was used for categorical variables.

To describe changes or differences in relative abundance of specific ARGs over time, a generalized linear mixed model (GLMM) for the negative binomial family with a log link was used to estimate the fold change in the group mean relative abundance during induction therapy. In this paper, the term 'fold change' is

used to describe the ratio of counts (not the exponent of the ratio of counts). Relative abundance of an ARG was calculated as the number of reads (rounded to integers) assigned to specific antibiotic resistance gene or class divided by the gene length and the total bacterial reads in the sample and is reported as 'reads per kilobase per million bacterial reads' (RPKM). The negative binomial GLMM with a log link included relative abundance of an ARG as response variables, prophylaxis group, time point and interaction between prophylaxis group and time point as covariates, and subject was treated as a random effect.

To describe changes or differences in the prevalence of specific ARGs over time, a binomial GLMM with a probit link with and without prophylaxis group effects was applied to estimate the prevalence of each gene or class at each time point during the ALL chemotherapy. A GLMM for binary outcomes (prevalence of an ARG) was applied, with a probit link, subject as random effect, and sample sequence depth ($\log_{10}$ total bacterial reads), prophylaxis group, time points, and interaction between prophylaxis group and time points as covariates. Prevalence was reported as the simple proportion of samples containing that gene or group. The GLMM probit binary model was also used to determine whether the estimated change in prevalence during induction therapy differed significantly between prophylaxis groups in the final analysis.

To describe the association between changes in ARGs and microbiome composition (relative abundance of specific phyla) over time, a linear mixed model (LMM) with subject as a random effect was applied. The $\log_{10}$ fold change, which was calculated as the RPKM of the ARGs at the end of the treatment phase + 1 divided by the RPKM of the ARGs at the start of the treatment phase + 1 was treated as the response variable. Prophylaxis group and change in phylum percentage relative abundance (defined as the compositional percentage of each phylum in a given microbiome sample) were included as covariates. To describe the association between changes in ARGs and antibiotic exposure, multivariate linear regression was performed with log10 fold change in ARG relative abundance as the response variable, and antibiotic exposure with either prophylaxis group or change in phylum percentage relative abundance as covariates.

2 Supplemental Tables

2.1 Dataset of characterized SNPs in topisomerase genes gyrA and parC associated with fluoroquinolone resistance

Gene	Gene Accession	Species	SNP mutation	Source DOI
gyrA	CP010781	Acinetobacter__baumannii	242 C->A	10.1128/aac.39.5.1201
gyrA	CP010781	Acinetobacter__baumannii	236 G->T	10.1128/aac.39.5.1201
gyrA	AF469609	Bartonella__bacilliformis	269 A->G	10.1128/AAC.47.1.383-386.2003
gyrA	AF469609	Bartonella__bacilliformis	283 G->A	10.1128/AAC.47.1.383-386.2003
gyrA	NC__002163	Campylobacter__jejuni	257 C->A	10.3389/fcimb.2012.00021
gyrA	NC__002163	Campylobacter__jejuni	257 C->T	10.3389/fcimb.2012.00021
gyrA	NC__002163	Campylobacter__jejuni	268 G->A	10.3389/fcimb.2012.00021
gyrA	ACLQ01000019	Capnocytophaga__gingivalis	238 G->A	10.1093/jac/dkx119
gyrA	ACLQ01000019	Capnocytophaga__gingivalis	245 C->T	10.1093/jac/dkx119
gyrA	ACLQ01000019	Capnocytophaga__gingivalis	245 C->A	10.1093/jac/dkx119
gyrA	ACLQ01000019	Capnocytophaga__gingivalis	242 A->G	10.1093/jac/dkx119
gyrA	NC__009089	Clostridium__difficile	245 C->T	10.3201/eid1303.060771
gyrA	U00096	Escherichia__coli	248 C->G	10.1128/AAC.42.10.2661
gyrA	U00096	Escherichia__coli	248 C->T	10.1128/AAC.42.10.2661
gyrA	U00096	Escherichia__coli	259 G->A	10.1128/AAC.42.10.2661
gyrA	U00096	Escherichia__coli	241 G->T	10.1128/AAC.42.10.2661
gyrA	U00096	Escherichia__coli	250 G->C	10.1128/AAC.42.10.2661
gyrA	NC__015964	Haemophilus__parainfluenza	251 C->T	10.1155/2010/525919
gyrA	NC__015964	Haemophilus__parainfluenza	262 G->T	10.1155/2010/525919
gyrA	NC__002516	Pseudomonas__aeruginosa	248 C->T	10.1128/AAC.39.9.1970
gyrA	NC__002516	Pseudomonas__aeruginosa	259 G->A	10.1128/AAC.39.9.1970
gyrA	NC__002516	Pseudomonas__aeruginosa	259 G->C	10.1128/AAC.39.9.1970
gyrA	NC__002516	Pseudomonas__aeruginosa	260 A->G	10.1128/AAC.39.9.1970
gyrA	CP003084	Cutibacterium__acnes	295 G->U	10.1016/j.anaerobe.2017.06.005
gyrA	CP003084	Cutibacterium__acnes	296 G->A	10.1016/j.anaerobe.2017.06.005
gyrA	CP003084	Cutibacterium__acnes	298 G->A	10.1016/j.anaerobe.2017.06.005
gyrA	CP003084	Cutibacterium__acnes	298 G->C	10.1016/j.anaerobe.2017.06.005
gyrA	CP003084	Cutibacterium__acnes	299 A->G	10.1016/j.anaerobe.2017.06.005
gyrA	CP003084	Cutibacterium__acnes	302 C->T	10.1016/j.anaerobe.2017.06.005
gyrA	CP003084	Cutibacterium__acnes	302 C->G	10.1016/j.anaerobe.2017.06.005
gyrA	CP003084	Cutibacterium__acnes	304 G->C	10.1016/j.anaerobe.2017.06.005
gyrA	CP003084	Cutibacterium__acnes	313 G->C	10.1016/j.anaerobe.2017.06.005
gyrA	CP003084	Cutibacterium__acnes	314 A->G	10.1016/j.anaerobe.2017.06.005
gyrA	NC__002946	Neisseria__gonorrhoeae	272 C->T	10.1128/JCM.42.12.5819-5824.2004
gyrA	NC__002946	Neisseria__gonorrhoeae	284 A->G	10.1128/JCM.42.12.5819-5824.2004
gyrA	NC__002946	Neisseria__gonorrhoeae	284 A->C	10.1128/JCM.42.12.5819-5824.2004
gyrA	NC__002946	Neisseria__gonorrhoeae	283 G->A	10.1128/JCM.42.12.5819-5824.2004
gyrA	AL450380	Mycobacterium__leprae	265 G->T	10.1128/AAC.01049-15
gyrA	AL450380	Mycobacterium__leprae	269 A->G	10.1128/AAC.01049-15
gyrA	AL450380	Mycobacterium__leprae	272 C->T	10.1128/AAC.01049-15
gyrA	AL450380	Mycobacterium__leprae	274 T->C	10.1128/AAC.01049-15
gyrA	AL450380	Mycobacterium__leprae	284 A->C	10.1128/AAC.01049-15
gyrB	NC__00952	Mycobacterium__tuberculosis	730 C->A	10.1128/AAC.01049-15
gyrB	NC__00952	Mycobacterium__tuberculosis	873 G->C	10.1128/AAC.01049-15
gyrB	NC__00952	Mycobacterium__tuberculosis	1207 G->T	10.1128/AAC.01049-15
gyrB	NC__00952	Mycobacterium__tuberculosis	1381 G->A	10.1128/AAC.01049-15
gyrB	NC__00952	Mycobacterium__tuberculosis	1453 A->G	10.1128/AAC.01049-15
gyrB	NC__00952	Mycobacterium__tuberculosis	1495 A->G	10.1128/AAC.01049-15
gyrB	NC__00952	Mycobacterium__tuberculosis	1496 A->C	10.1128/AAC.01049-15

Gene	Gene Accesion	Species	SNP mutation	Source DOI
gyrB	NC__00952	Mycobacterium__tuberculosis	1522 G->T	10.1128/AAC.01049-15
gyrB	NC__00952	Mycobacterium__tuberculosis	1534 G->T	10.1128/AAC.01049-15
gyrA	AL123456	Mycobacterium__tuberculosis	262 G->T	10.1128/AAC.01049-15
gyrA	AL123456	Mycobacterium__tuberculosis	266 A->G	10.1128/AAC.01049-15
gyrA	AL123456	Mycobacterium__tuberculosis	269 C->T	10.1128/AAC.01049-15
gyrA	AL123456	Mycobacterium__tuberculosis	271 T->C	10.1128/AAC.01049-15
gyrA	AL123456	Mycobacterium__tuberculosis	281 A->C	10.1128/AAC.01049-15
gyrB	AL450380	Mycobacterium__leprae	1605 C->T	10.1371/journal.pntd.0001838
gyrB	AL450380	Mycobacterium__leprae	1603 G->C	10.1371/journal.pntd.0001838
gyrB	AL450380	Mycobacterium__leprae	1604 A->C	10.1371/journal.pntd.0001838
gyrB	AL450380	Mycobacterium__leprae	1678 A->C	10.1371/journal.pntd.0001838
gyrB	NC__020418	Morganella__morganii	1478 T->G	10.1186/s12941-014-0034-4
gyrB	NC__020418	Morganella__morganii	1481 T->G	10.1186/s12941-014-0034-4
gyrB	NC__020418	Morganella__morganii	1538 T->G	10.1186/s12941-014-0034-4
gyrB	NC__020418	Morganella__morganii	1482 C->A	10.1186/s12941-014-0034-4
gyrB	NC__020418	Morganella__morganii	1485 C->A	10.1186/s12941-014-0034-4
gyrB	NC__020418	Morganella__morganii	1542 C->A	10.1186/s12941-014-0034-4
gyrB	NC__003197	Salmonella__enterica	1726 C->A	10.1093/cid/civ790
gyrB	NC__003197	Salmonella__enterica	1734 A->C	10.1093/cid/civ790
gyrB	NC__003197	Salmonella__enterica	1734 A->T	10.1093/cid/civ790
gyrA	NC__004337	Shigella__flexneri	248 C->T	10.1007/s00284-005-0140-9
gyrA	NC__004337	Shigella__flexneri	259 G->A	10.1007/s00284-005-0140-9
gyrA	NC__004337	Shigella__flexneri	260 A->G	10.1007/s00284-005-0140-9
gyrA	NC__004337	Shigella__flexneri	241 A->G	10.1007/s00284-005-0140-9
gyrA	NC__004337	Shigella__flexneri	275 T->A	10.1007/s00284-005-0140-9
gyrA	NC__002952	Staphylococcus__aureus	251 C->T	10.1128/AAC.42.5.1249
gyrA	NC__002952	Staphylococcus__aureus	253 C->T	10.1128/AAC.42.5.1249
gyrA	NC__002952	Staphylococcus__aureus	262 G->A	10.1128/AAC.42.5.1249
gyrB	NC__011374	Ureaplasma__urealyticum	355 T->C	10.1128/AAC.06342-11
gyrB	NC__011374	Ureaplasma__urealyticum	1384 C->T	10.1128/AAC.06342-11
gyrB	AP009048	Escherichia	1240 G->A	10.1128/AAC.42.5.1249
gyrB	AP009048	Escherichia	1296 A->G	10.1128/AAC.42.5.1249
gyrA	CP001918	Enterobacter__cloacae	248 C->T	10.1093/jac/40.4.543
gyrA	CP001918	Enterobacter__cloacae	248 C->A	10.1093/jac/40.4.543
gyrA	CP001918	Enterobacter__cloacae	250 G->C	10.1093/jac/40.4.543
gyrA	CP001918	Enterobacter__cloacae	259 G->C	10.1093/jac/40.4.543
gyrA	CP001918	Enterobacter__cloacae	259 G->A	10.1093/jac/40.4.543
gyrA	CP001918	Enterobacter__cloacae	260 A->G	10.1093/jac/40.4.543
gyrA	CP001918	Enterobacter__cloacae	260 A->T	10.1093/jac/40.4.543
gyrA	CP001918	Enterobacter__cloacae	260 A->C	10.1093/jac/40.4.543
gyrA	NC__008702	Azoarcus	242 G->T	10.1128/JCM.41.7.3273-3283.2003
gyrA	NC__008702	Azoarcus	248 C->T	10.1128/JCM.41.7.3273-3283.2003
gyrA	NC__009725	Bacillus__amyloliquefaciens	173 C->T	10.1128/genomeA.00633-14
gyrA	NC__009725	Bacillus__amyloliquefaciens	184 G->A	10.1128/genomeA.00633-14
gyrA	CP000647	Klebsiella__pneumoniae	248 C->A	10.1128/AAC.41.3.699
gyrA	CP000647	Klebsiella__pneumoniae	248 C-> T	10.1128/AAC.41.3.699
gyrA	CP000647	Klebsiella__pneumoniae	259 G->A	10.1128/AAC.41.3.699
gyrA	CP000647	Klebsiella__pneumoniae	260 A->G	10.1128/AAC.41.3.699
gyrA	HE999704	Listeria__monocytogenes	250 T->A	10.1093/jac/dkf065
gyrA	HE999704	Listeria__monocytogenes	263 C->T	10.1093/jac/dkf065
gyrA	CP000675	Legionella__pneumophila	248 C->T	10.1093/jac/dkp173
gyrA	CP000675	Legionella__pneumophila	248 C->A	10.1093/jac/dkp173
gyrA	CP000675	Legionella__pneumophila	259 G->A	10.1093/jac/dkp173
gyrA	CP000308	Yersinia__pestis	241 G->T	10.1128/JCM.41.7.3273-3283.2003
gyrA	CP000308	Yersinia__pestis	242 G->A	10.1128/JCM.41.7.3273-3283.2003

Gene	Gene Accesion	Species	SNP mutation	Source DOI
gyrA	CP000308	Yersinia__pestis	248 G->T	10.1128/JCM.41.7.3273-3283.2003
gyrA	CP000308	Yersinia__pestis	249 C->A	10.1128/JCM.41.7.3273-3283.2003
gyrA	CP000308	Yersinia__pestis	249 C->G	10.1128/JCM.41.7.3273-3283.2003
gyrA	CP002332	Helicobacter__pylori	260 C->T	10.1128/AAC.05243-11
gyrA	CP002332	Helicobacter__pylori	261 C->A	10.1128/AAC.05243-11
gyrA	CP002332	Helicobacter__pylori	261 C->G	10.1128/AAC.05243-11
gyrA	CP002332	Helicobacter__pylori	262 G->C	10.1128/AAC.05243-11
gyrA	CP002332	Helicobacter__pylori	272 A->G	10.1128/AAC.05243-11
gyrA	CP002332	Helicobacter__pylori	271 G->A	10.1128/AAC.05243-11
gyrA	CP002332	Helicobacter__pylori	271 G->C	10.1128/AAC.05243-11
gyrA	CP002332	Helicobacter__pylori	271 G->T	10.1128/AAC.05243-11
gyrA	FQ312006	Haemophilus__influenzae	251 C->T	10.1128/AAC.40.7.1741
gyrA	FQ312006	Haemophilus__influenzae	251 C->A	10.1128/AAC.40.7.1741
gyrA	FQ312006	Haemophilus__influenzae	262 G->A	10.1128/AAC.40.7.1741
gyrA	FQ312006	Haemophilus__influenzae	262 G->T	10.1128/AAC.40.7.1741
gyrA	NC__012469	Streptococcus__pneumoniae	242 C->T	10.1128/AAC.40.12.2760
gyrA	NC__012469	Streptococcus__pneumoniae	242 C->A	10.1128/AAC.40.12.2760
gyrA	NC__012469	Streptococcus__pneumoniae	253 G->A	10.1128/AAC.40.12.2760
gyrA	CP004022	Proteus__mirabilis	247 A->C	10.1128/AAC.46.8.2582-2587.2002
gyrA	CP004022	Proteus__mirabilis	248 G->T	10.1128/AAC.46.8.2582-2587.2002
gyrA	CP004022	Proteus__mirabilis	259 G->A	10.1128/AAC.46.8.2582-2587.2002
gyrA	NC__006582	Bacillus__clausii	254 C->U	10.1007/s10156-012-0532-2
gyrA	NC__006582	Bacillus__clausii	304 A->C	10.1007/s10156-012-0532-2
gyrA	NC__006582	Bacillus__clausii	205 T->C	10.1007/s10156-012-0532-2
gyrA	CP002695	Bordetella__pertussis	260 A->G	10.1128/AAC.00023-09
gyrA	AP010904	Desulfovibrio__magneticus	235 G->A	10.1007/s10156-012-0532-2
gyrA	AP010904	Desulfovibrio__magneticus	240 A->G	10.1007/s10156-012-0532-2
gyrA	CP000034	Shigella__dysenteriae	248 C->T	10.1016/j.ijid.2017.03.023
gyrA	CP000034	Shigella__dysenteriae	259 G->A	10.1016/j.ijid.2017.03.023
gyrA	CP000034	Shigella__dysenteriae	260 A->G	10.1016/j.ijid.2017.03.023
gyrA	AL009126	Bacillus__subtilis	250 T->G	10.1007/bf00280192
gyrA	AL009126	Bacillus__subtilis	251 C->T	10.1007/bf00280192
gyrA	AL009126	Bacillus__subtilis	253 G->C	10.1007/bf00280192
gyrA	AL009126	Bacillus__subtilis	262 G->A	10.1007/bf00280192
gyrA	AL009126	Bacillus__subtilis	262 G->C	10.1007/bf00280192
gyrA	AL009126	Bacillus__subtilis	263 A->G	10.1007/bf00280192
gyrA	AL009126	Bacillus__subtilis	263 A->T	10.1007/bf00280192
gyrA	NC__012491	Brevibacillus__brevis	248 C->T	10.1007/s10156-012-0532-2
gyrA	NC__012491	Brevibacillus__brevis	265 A->T	10.1007/s10156-012-0532-2
gyrA	NC__012491	Brevibacillus__brevis	267 G->T	10.1007/s10156-012-0532-2
gyrA	NC__012491	Brevibacillus__brevis	267 G->C	10.1007/s10156-012-0532-2
gyrA	NC__012491	Brevibacillus__brevis	267 G->A	10.1007/s10156-012-0532-2
gyrA	NC__012491	Brevibacillus__brevis	301 A->C	10.1007/s10156-012-0532-2
gyrA	NC__012491	Brevibacillus__brevis	302 A->C	10.1007/s10156-012-0532-2
gyrA	CP003275	Streptomyces__hygroscopicus	277 G->A	10.1039/c3mb70341j
gyrA	CP003275	Streptomyces__hygroscopicus	278 G->A	10.1039/c3mb70341j
gyrA	CP003275	Streptomyces__hygroscopicus	281 A->G	10.1039/c3mb70341j
gyrA	CP003275	Streptomyces__hygroscopicus	284 C->T	10.1039/c3mb70341j
gyrA	CP003275	Streptomyces__hygroscopicus	284 C->A	10.1039/c3mb70341j
gyrA	CP001581	Clostridium__botulinum	245 C->T	10.1128/AAC.49.2.488-492.2005
gyrA	AE016830	Enterococcus__faecalis	250 A->C	10.1016/S0378-1097(03)00929-7
gyrA	AE016830	Enterococcus__faecalis	251 G->T	10.1016/S0378-1097(03)00929-7
gyrA	AE016830	Enterococcus__faecalis	262 G->A	10.1016/S0378-1097(03)00929-7
gyrA	AE016830	Enterococcus__faecalis	263 A->G	10.1016/S0378-1097(03)00929-7
gyrA	CP001485	Vibrio__cholerae	248 G->T	10.1016/j.ijantimicag.2013.03.004

Gene	Gene Accesion	Species	SNP mutation	Source DOI
gyrA	CP001485	Vibrio_cholerae	259 G->A	10.1016/j.ijantimicag.2013.03.004
gyrA	NC_003112	Neisseria_meningitidis	272 C->T	10.1093/jac/dkm452
gyrA	NC_003112	Neisseria_meningitidis	283 G->A	10.1093/jac/dkm452
gyrA	NC_003112	Neisseria_meningitidis	307 A->G	10.1093/jac/dkm452
gyrA	AE015929	Staphylococcus_epidermidis	250 C->T	10.1136/bjo.2007.129858
gyrA	AE015929	Staphylococcus_epidermidis	250 C->A	10.1136/bjo.2007.129858
gyrA	AE015929	Staphylococcus_epidermidis	262 A->G	10.1136/bjo.2007.129858
parC	AB003428	Pseudomonas_aeruginosa	260 C->T	10.1007/s10156-012-0455-y
parC	AB003428	Pseudomonas_aeruginosa	273 G->C	10.1007/s10156-012-0455-y
parC	AB003428	Pseudomonas_aeruginosa	275 C->G	10.1007/s10156-012-0455-y
parC	CP012952	Acinetobacter_baumannii	251 C->T	PMID:ă26221488
parC	CP012952	Acinetobacter_baumannii	262 G->A	PMID:ă26221488
parC	CP012952	Acinetobacter_baumannii	244 G->T	PMID:ă26221488
parC	U00096	Escherichia_coli	239 G->T	10.1128/JCM.01093-07
parC	U00096	Escherichia_coli	250 G->A	10.1128/JCM.01093-07
parC	U00096	Escherichia_coli	240 C->A	10.1128/JCM.01093-07
parC	U00096	Escherichia_coli	240 C->G	10.1128/JCM.01093-07
parC	U00096	Escherichia_coli	251 A->T	10.1128/JCM.01093-07
parC	U00096	Escherichia_coli	251 A->G	10.1128/JCM.01093-07
parC	U00096	Escherichia_coli	323 C->T	10.1128/JCM.01093-07
parC	NC_007779	Escherichia_coli	1372 T_>G	10.1128/JCM.01093-07
parC	NC_007779	Escherichia_coli	1278 C_>A	10.1128/JCM.01093-07
parC	NC_015964	Haemophilus_parainfluenzae	251 C->T	10.1155/2010/525919
parC	CP011538	Mycoplasma_hominis	255 T_>C	10.1128/AAC.43.4.954
parC	CP011538	Mycoplasma_hominis	264 T->G	10.1128/AAC.43.4.954
parC	AE005672	Streptococcus_pneumoniae	239 C->A	10.1128/AAC.40.12.2760
parC	AE005672	Streptococcus_pneumoniae	239 C->T	10.1128/AAC.40.12.2760
parC	AE005672	Streptococcus_pneumoniae	248 A->G	10.1128/AAC.40.12.2760
parC	NC_002952	Staphylococcus_aureus	1357 C->T	10.1128/AAC.42.11.3044
parC	NC_002952	Staphylococcus_aureus	1299 T_>C	10.1128/AAC.42.11.3044
parC	NC_002952	Staphylococcus_aureus	1300 G->C	10.1128/AAC.42.11.3044
parC	NC_002952	Staphylococcus_aureus	1300 G->A	10.1128/AAC.42.11.3044
parC	NC_002952	Staphylococcus_aureus	1301 A->G	10.1128/AAC.42.11.3044
parC	NC_002952	Staphylococcus_aureus	1414 A->G	10.1128/AAC.42.11.3044
parC	NC_002952	Staphylococcus_aureus	239 C->T	10.1128/AAC.42.5.1249
parC	NC_002952	Staphylococcus_aureus	251 A->T	10.1128/AAC.42.5.1249
parC	NC_002952	Staphylococcus_aureus	142 G->A	10.1128/AAC.42.5.1249
parC	NC_004337	Shigella_flexneri	239 G->T	10.1016/j.ijid.2017.03.023
parC	NC_004337	Shigella_flexneri	256 A->T	10.1016/j.ijid.2017.03.023
parC	NC_004337	Shigella_flexneri	257 T->G	10.1016/j.ijid.2017.03.023
parC	NC_004337	Shigella_flexneri	385 T->C	10.1016/j.ijid.2017.03.023
parC	NC_003197	Salmonella_enterica	250 G->A	10.3201/eid0911.030317
parC	NC_003197	Salmonella_enterica	240 C->A	10.3201/eid0911.030317
parC	NC_003197	Salmonella_enterica	240 C->G	10.3201/eid0911.030317
parC	NC_002516	Pseudomonas_aeruginosa	1166 G->A	10.1128/AAC.39.9.1970
parC	NC_002516	Pseudomonas_aeruginosa	1369 A->G	10.1128/AAC.39.9.1970
parC	NC_002516	Pseudomonas_aeruginosa	1376 A->T	10.1128/AAC.39.9.1970
parC	NC_002516	Pseudomonas_aeruginosa	1328 C->T	10.1128/AAC.39.9.1970
parC	NC_002516	Pseudomonas_aeruginosa	1378 G->T	10.1128/AAC.39.9.1970
parC	NC_002946	Neisseria_gonorrhoeae	256 G->A	10.1128/JCM.42.12.5819-5824.2004
parC	NC_002946	Neisseria_gonorrhoeae	259 A->C	10.1128/JCM.42.12.5819-5824.2004
parC	NC_002946	Neisseria_gonorrhoeae	260 G->A	10.1128/JCM.42.12.5819-5824.2004
parC	NC_002946	Neisseria_gonorrhoeae	260 G->T	10.1128/JCM.42.12.5819-5824.2004
parC	NC_002946	Neisseria_gonorrhoeae	261 T->G	10.1128/JCM.42.12.5819-5824.2004
parC	NC_002946	Neisseria_gonorrhoeae	271 G->A	10.1128/JCM.42.12.5819-5824.2004

Gene	Gene Accesion	Species	SNP mutation	Source DOI
parC	NC_002946	Neisseria_gonorrhoeae	271 G->C	10.1128/JCM.42.12.5819-5824.2004
parC	NC_002946	Neisseria_gonorrhoeae	272 A->G	10.1128/JCM.42.12.5819-5824.2004
parC	NC_002946	Neisseria_gonorrhoeae	272 A->C	10.1128/JCM.42.12.5819-5824.2004
parC	NC_002162	Ureaplasma_urealyticum	248 C->T	10.1128/AAC.06342-11
parC	NC_002162	Ureaplasma_urealyticum	259 G->A	10.1128/AAC.06342-11
parC	NC_002162	Ureaplasma_urealyticum	8 T->A	10.1128/AAC.06342-11
parC	NC_012491	Brevibacillus_brevis	253 G->A	10.1128/AAC.47.7.2362-2365.2003
parC	NC_011586	Acinetobacter_baumannii	251 C->T	PMID:ă26221488
parC	NC_011586	Acinetobacter_baumannii	262 G->A	PMID:ă26221488
parC	NC_011586	Acinetobacter_baumannii	244 G->T	PMID:ă26221488
parC	AE016830	Enterococcus_faecalis	245 G->T	10.1128/AAC.43.4.947
parC	CP004022	Proteus_mirabilis	245 G->A	10.1128/AAC.46.8.2582-2587.2002
parC	CP004022	Proteus_mirabilis	251 G->T	10.1128/AAC.46.8.2582-2587.2002
parC	CP004022	Proteus_mirabilis	252 C->A	10.1128/AAC.46.8.2582-2587.2002
parC	CP004022	Proteus_mirabilis	252 C->G	10.1128/AAC.46.8.2582-2587.2002
parC	CP000034	Shigella_dysenteriae	239 G->T	10.1016/j.ijid.2017.03.023
parC	CP000034	Shigella_dysenteriae	256 A->T	10.1016/j.ijid.2017.03.023
parC	CP000034	Shigella_dysenteriae	257 T->G	10.1016/j.ijid.2017.03.023
parC	CP000034	Shigella_dysenteriae	385 T->C	10.1016/j.ijid.2017.03.023
parC	CP001581	Clostridium_botulinum	275 A->T	10.1128/AAC.49.2.488-492.2005
parC	NC_003112	Neisseria_meningitidis	256 G->A	10.1128/JCM.42.12.5819-5824.2004
parC	NC_003112	Neisseria_meningitidis	259 A->C	10.1128/JCM.42.12.5819-5824.2004
parC	NC_003112	Neisseria_meningitidis	260 G->A	10.1128/JCM.42.12.5819-5824.2004
parC	NC_003112	Neisseria_meningitidis	260 G->T	10.1128/JCM.42.12.5819-5824.2004
parC	NC_003112	Neisseria_meningitidis	261 T->G	10.1128/JCM.42.12.5819-5824.2004
parC	NC_003112	Neisseria_meningitidis	271 G->A	10.1128/JCM.42.12.5819-5824.2004
parC	NC_003112	Neisseria_meningitidis	271 G->C	10.1128/JCM.42.12.5819-5824.2004
parC	NC_003112	Neisseria_meningitidis	272 A->G	10.1128/JCM.42.12.5819-5824.2004
parC	NC_003112	Neisseria_meningitidis	272 A->C	10.1128/JCM.42.12.5819-5824.2004
parC	CR628337	Legionella_pneumophila	251 G->A	10.1093/jac/dkp173
parC	CR628337	Legionella_pneumophila	256 A->C	10.1093/jac/dkp173
parC	CP003583	Enterococcus_faecium	245 G->T	10.1128/AAC.43.4.947
parC	NC_006582	Bacillus_clausii	239 C->A	10.1007/s10156-012-0532-2
parC	NC_006582	Bacillus_clausii	239 C->T	10.1007/s10156-012-0532-2

2.2 Comparison of characteristics of included with excluded participants

	Included (n=49)		Excluded (n=62)		
Characteristic	n/N	(%)	n/N	(%)	P value
**Age**, median (range)	6.9	(0.8–17.8)	5.9	(1.4 –18)	0.45
**Sex**					0.56
Female	21/49	(42.9%)	23/62	(37.1%)	
Male	28/49	(57.1%)	39/62	(62.9%)	
**Race**					0.44
White	43/49	(87.8%)	50/62	(80.6%)	
Black	6/49	(12.2%)	10/62	(16.1%)	
Other	0/49	(0%)	2/62	(3.2%)	
**Leukemia risk category**					0.96
Low	20/49	(40.8%)	27/62	(43.5%)	
Standard	25/49	(51%)	30/62	(48.4%)	
High	4/49	(8.2%)	5/62	(8.1%)	
**Prophylaxis regimen**					0.18
None	18/49	(36.7%)	31/62	(50%)	
Levofloxacin	31/49	(63.3%)	31/62	(50%)	

Table 2: Characteristics of included participants compared with eligible participants excluded because of non-availability of samples. P-values estimated by Fisher's exact test for dichotomous variables and by Mann-Whitney U test for continuous variables.

2.3 Antibiotic susceptibility patterns in isolates from bloodstream infection

	Levofloxacin Proph.		No Prophylaxis	
Antibiotic	n/N	% Susceptible	n/N	% Susceptible
**Gram positive organisms**	6 isolates		3 isolates	
Levofloxacin	1/5	20%	0/2	0%
Methicillin	1/5	20%	0/3	0%
Clindamycin	2/3	67%	2/2	100%
**Gram negative organisms**	3 isolates		3 isolates	
Levofloxacin	3/3	100%	2/3	67%
Cefepime	3/3	100%	3/3	100%

Table 3: Proportion of isolates from blood cultures that were susceptible to levofloxacin did not differ between groups. n/N is the number of susceptible isolates/number tested. Antibiotic resistance testing is done based on species appropriateness.

2.4 Comparison of sequencing depth between prophylaxis groups

		Total Bacterial Reads			Total Sequenced Reads		
Prophylaxis	Timepoint	Median	Range	P value	Median	Range	P value
No prophylaxis	Baseline	4.2×10^5	8.9×10^4–1.1×10^6	0.20	1.7×10^6	5.3×10^5–7.0×10^6	0.43
Levofloxacin	Baseline	5.7×10^5	1.4×10^5–2.7×10^6		2.3×10^6	7.5×10^5–9.1×10^6	
No prophylaxis	Post-Induction	4.4×10^5	1.5×10^5–1.3×10^6	0.25	1.9×10^6	8.0×10^5–1.0×10^7	0.85
Levofloxacin	Post-Induction	4.6×10^5	9.1×10^4–2.0×10^6		2.3×10^6	7.3×10^5–7.7×10^6	
No prophylaxis	Post-Consolidation	4.4×10^5	7.9×10^4–6.2×10^6	0.49	2.1×10^6	4.2×10^5–8.3×10^6	0.99
Levofloxacin	Post-Consolidation	4.4×10^5	4.3×10^4–2.0×10^6		2.2×10^6	4.4×10^5–5.3×10^6	

Table 4: Total bacterial reads and total sequencing reads varied between samples but were not significantly different between prophylactic groups. P values are for two-way t-test of depth of bacterial and total sequenced reads between prophylactic groups. Total bacterial reads as determined via Kraken classification varied from total sequenced reads due to host, fungal and other DNA being present in stool sample extraction.

2.5 Detection frequency of ARGs among 118 samples

**Gene**	**Class**	**Mechanism**	**n**	**frequency**
dfrF	Sul-Tri	dihydrofolate reductase	105	0.89
CfxA2	Beta-lactam (SA2)	class A beta-lactamase	98	0.83
tetW	Tetracycline	ribosomal protection protein	92	0.78
tetO	Tetracycline	ribosomal protection protein	77	0.65
ErmF	MLS	erm 23S rRNA methyltransferase	67	0.57
tetX	Tetracycline	flavin-dependent monooxygenase	67	0.57
tetQ	Tetracycline	ribosomal protection protein	58	0.49
ErmB	MLS	erm 23S rRNA methyltransferase	53	0.45
tet32	Tetracycline	ribosomal protection protein	47	0.4
ErmG	MLS	erm 23S rRNA methyltransferase	40	0.34
CblA-1	Beta-lactam (SA2)	class A beta-lactamase	39	0.33
APH(3')-IIIa	Aminoglycoside	aminoglycoside phosphotransferase	37	0.31
CfxA3	Beta-lactam (SA2)	class A beta-lactamase	37	0.31
CfxA6	Beta-lactam (SA2)	class A beta-lactamase	24	0.2
SAT-4	Streptothricin	streptothricin acetyltransferase	24	0.2
tet40	Tetracycline	tetracycline efflux pump	21	0.18
lnuC	MLS	nucleotidyltransferase	20	0.17
mel	MLS	macrolide efflux pump	20	0.17
sul2	Sul-Tri	sulfonamide resistant dihydropteroate synthase	18	0.15
ErmX	MLS	erm 23S rRNA methyltransferase	16	0.14
aad(6)	Aminoglycoside	aminoglycoside nucleotidyltransferase	14	0.12
ANT(6)-Ia	Aminoglycoside	aminoglycoside nucleotidyltransferase	14	0.12
tetB	Tetracycline	ribosomal protection protein	14	0.12
APH(3")-Ib	Aminoglycoside	aminoglycoside phosphotransferase	12	0.1
TEM-126	Beta-lactam (SA1)	class A beta-lactamase	12	0.1
AAC(6')-Ii	Aminoglycoside	aminoglycoside acetyltransferase	11	0.09
APH(6)-Id	Aminoglycoside	aminoglycoside phosphotransferase	11	0.09
ErmQ	MLS	erm 23S rRNA methyltransferase	11	0.09
cepA	Beta-lactam (SA2)	class A beta-lactamase	10	0.08
H-NS	Unclassified	resistance associated regulatory genes	9	0.08
marA	Multidrug	multidrug efflux pump	9	0.08
CRP	Multidrug	multidrug efflux pump	8	0.07
dfrA14	Sul-Tri	dihydrofolate reductase	8	0.07

Gene	Class	Mechanism	n	frequency
emrR	Multidrug	multidrug efflux pump	8	0.07
gadW	Multidrug	multidrug efflux pump	8	0.07
Mrx	MLS	macrolide inactivation enzyme	8	0.07
tetD	Tetracycline	tetracycline efflux pump	8	0.07
AAC(6')-Ie-APH(2")-Ia	Aminoglycoside	aminoglycoside acetyltransferase	7	0.06
ACI-1	Beta-lactam (SA1)	class A beta-lactamase	7	0.06
acrA	Multidrug	multidrug efflux pump	7	0.06
APH(2")-IIa	Aminoglycoside	aminoglycoside phosphotransferase	7	0.06
efmA	Multidrug	multidrug efflux pump	7	0.06
gyrA	Quinolone	topoisomerase point mutation	7	0.06
CdeA	Multidrug	multidrug efflux pump	6	0.05
emrB	Multidrug	multidrug efflux pump	6	0.05
emrK	Multidrug	multidrug efflux pump	6	0.05
evgA	Multidrug	multidrug efflux pump	6	0.05
lsaC	MLS	ribosomal protection protein	6	0.05
mphA	MLS	macrolide inactivation enzyme	6	0.05
tolC	Multidrug	multidrug efflux pump	6	0.05
acrB	Multidrug	multidrug efflux pump	5	0.04
acrD	Aminoglycoside	aminoglycoside efflux pump	5	0.04
CfxA4	Beta-lactam (SA2)	class A beta-lactamase	5	0.04
cpxA	Multidrug	multidrug efflux pump	5	0.04
dfrA17	Sul-Tri	dihydrofolate reductase	5	0.04
efrA	Multidrug	multidrug efflux pump	5	0.04
emeA	Multidrug	multidrug efflux pump	5	0.04
emrA	Multidrug	multidrug efflux pump	5	0.04
evgS	Unclassified	resistance associated regulatory genes	5	0.04
mdtC	Multidrug	multidrug efflux pump	5	0.04
mdtE	Multidrug	multidrug efflux pump	5	0.04
mdtN	Multidrug	multidrug efflux pump	5	0.04
parC	Quinolone	topoisomerase point mutation	5	0.04
patA	Quinolone	quinolone-specific efflux pump	5	0.04
sul1	Sul-Tri	sulfonamide resistant dihydropteroate synthase	5	0.04
acrF	Multidrug	multidrug efflux pump	4	0.03
bacA	Bacitracin	undecaprenyl pyrophosphate recycle	4	0.03
baeS	Unclassified	resistance associated regulatory genes	4	0.03
emrY	Multidrug	multidrug efflux pump	4	0.03
eptA	Polymixin	phosphoethanolamine transferase	4	0.03
mefA	MLS	macrolide efflux pump	4	0.03
pmrF	Polymixin	phosphoethanolamine transferase	4	0.03
tetA(P)	Tetracycline	tetracycline efflux pump	4	0.03
vanHA	Vancomycin	terminal peptidoglycan synthesis	4	0.03
vanSA	Vancomycin	regulates peptidoglycan	4	0.03
vanXA	Vancomycin	regulates peptidoglycan	4	0.03
vanZA	Vancomycin	accessory protein	4	0.03
aadA5	Aminoglycoside	aminoglycoside nucleotidyltransferase	3	0.03
acrE	Multidrug	multidrug efflux pump	3	0.03
ampC	Beta-lactam (SC)	class C beta-lactamase	3	0.03
baeR	Multidrug	multidrug efflux pump	3	0.03
CfxA5	Beta-lactam (SA2)	class A beta-lactamase	3	0.03
efrB	Multidrug	multidrug efflux pump	3	0.03
ileS	Mupirocin	tRNA synthetase	3	0.03
mdtB	Multidrug	multidrug efflux pump	3	0.03
mdtG	Multidrug	multidrug efflux pump	3	0.03
mdtO	Multidrug	multidrug efflux pump	3	0.03
msbA	Multidrug	multidrug efflux pump	3	0.03

Gene	Class	Mechanism	n	frequency
tet44	Tetracycline	ribosomal protection protein	3	0.03
tetM	Tetracycline	ribosomal protection protein	3	0.03
vanA	Vancomycin	terminal peptidoglycan synthesis	3	0.03
vanRC	Vancomycin	regulates peptidoglycan	3	0.03
vanYA	Vancomycin	regulates peptidoglycan	3	0.03
acrS	Multidrug	multidrug efflux pump	2	0.02
ANT(6)-Ib	Aminoglycoside	aminoglycoside nucleotidyltransferase	2	0.02
ANT(9)-Ia	Aminoglycoside	aminoglycoside nucleotidyltransferase	2	0.02
APH(2")-IVa	Aminoglycoside	aminoglycoside phosphotransferase	2	0.02
APH(3')-Ia	Aminoglycoside	aminoglycoside phosphotransferase	2	0.02
cat	Chloramphenicol	Chloramphenicol acetyltransferase variants	2	0.02
dfrC	Sul-Tri	dihydrofolate reductase	2	0.02
dfrG	Sul-Tri	dihydrofolate reductase	2	0.02
gadX	Multidrug	multidrug efflux pump	2	0.02
kdpE	Unclassified	resistance associated regulatory genes	2	0.02
MCR-5	Polymixin	phosphoethanolamine transferase	2	0.02
mdtA	Multidrug	multidrug efflux pump	2	0.02
mdtF	Multidrug	multidrug efflux pump	2	0.02
mdtH	Multidrug	multidrug efflux pump	2	0.02
mdtM	Multidrug	multidrug efflux pump	2	0.02
mdtP	Multidrug	multidrug efflux pump	2	0.02
qacA	Multidrug	multidrug efflux pump	2	0.02
TEM-34	Beta-lactam (SA1)	class A beta-lactamase	2	0.02
tetA	Tetracycline	tetracycline efflux pump	2	0.02
vanC	Vancomycin	terminal peptidoglycan synthesis	2	0.02
vanRA	Vancomycin	regulates peptidoglycan	2	0.02
vanSC	Vancomycin	regulates peptidoglycan	2	0.02
vanTC	Vancomycin	regulates peptidoglycan	2	0.02
vanXYC	Vancomycin	regulates peptidoglycan	2	0.02
yojI	Multidrug	multidrug efflux pump	2	0.02
AAC(6')-Ib-cr	Aminoglycoside	aminoglycoside acetyltransferase	1	0.01
ANT(4')-Ib	Aminoglycoside	aminoglycoside nucleotidyltransferase	1	0.01
APH(2")-If	Aminoglycoside	aminoglycoside phosphotransferase	1	0.01
APH(2")-IIIa	Aminoglycoside	aminoglycoside phosphotransferase	1	0.01
catB3	Chloramphenicol	Chloramphenicol acetyltransferase variants	1	0.01
catP	Chloramphenicol	Chloramphenicol acetyltransferase variants	1	0.01
CTX-M-155	Beta-lactam (SA1)	class A beta-lactamase	1	0.01
dfrA1	Sul-Tri	dihydrofolate reductase	1	0.01
dfrA16	Sul-Tri	dihydrofolate reductase	1	0.01
dfrA5	Sul-Tri	dihydrofolate reductase	1	0.01
emrE	Multidrug	multidrug efflux pump	1	0.01
ErmC	MLS	erm 23S rRNA methyltransferase	1	0.01
ErmT	MLS	erm 23S rRNA methyltransferase	1	0.01
floR	Chloramphenicol	Chloramphenicol efflux pump	1	0.01
lmrC	MLS	macrolide efflux pump	1	0.01
lnuB	MLS	nucleotidyltransferase	1	0.01
lnuG	MLS	nucleotidyltransferase	1	0.01
lsaA	MLS	ribosomal protection protein	1	0.01
lsaE	MLS	macrolide efflux pump	1	0.01
mdfA	Multidrug	multidrug efflux pump	1	0.01
mecA	Beta-lactam (PBP)	penicillin-binding protein	1	0.01
msrA	MLS	ribosomal protection protein	1	0.01
msrC	MLS	ribosomal protection protein	1	0.01
norA	Multidrug	multidrug efflux pump	1	0.01
OKP-B-3	Beta-lactam (SA1)	class A beta-lactamase	1	0.01

Gene	Class	Mechanism	n	frequency
OXA-31	Beta-lactam (SD2)	class D beta-lactamase	1	0.01
OXA-347	Beta-lactam (SD1)	class D beta-lactamase	1	0.01
pmrE	Polymixin	phosphoethanolamine transferase	1	0.01
QnrB19	Quinolone	DNA gyrase modifier	1	0.01
QnrB47	Quinolone	DNA gyrase modifier	1	0.01
QnrB5	Quinolone	DNA gyrase modifier	1	0.01
QnrB62	Quinolone	DNA gyrase modifier	1	0.01
SAT-1	Streptothricin	streptothricin acetyltransferase	1	0.01
TEM-59	Beta-lactam (SA1)	class A beta-lactamase	1	0.01
tetL	Tetracycline	tetracycline efflux pump	1	0.01
vanHD	Vancomycin	terminal peptidoglycan synthesis	1	0.01
vanRB	Vancomycin	regulates peptidoglycan	1	0.01
vanRD	Vancomycin	regulates peptidoglycan	1	0.01
vanYD	Vancomycin	regulates peptidoglycan	1	0.01

Table 5: Only a few specific antibiotic resistance genes were common. with the majority of those identified in this cohort occurring in less than 50% of the population. For genes or ARG classes that were present in at least 50% of samples, the primary comparison was relative abundance between groups. For genes or ARG classes that were less common prevalence was the primary comparator between groups. MLS refers to macrolide, lincosamide and streptogramin antibiotics. Sul-Tri refers to Sulfamethoxazole-Trimethoprim.

2.6 Estimated prevalence of antibiotic resistance gene classes at each time point based on GLMMs for binary outcomes

ARG	Sample	Levofloxacin	No prophylaxis	All
Aminoglycoside	Baseline	26.71% (10.25%. 50.96%)	46.24% (18.3%. 76.27%)	39.35% (24.74%. 55.65%)
Aminoglycoside	Post-induction	34.53% (15.27%. 59.05%)	66.42% (36.17%. 88.53%)	46.44% (31.4%. 62.01%)
Aminoglycoside	Post-consolidation	14.86% (3.55%. 39.02%)	69.14% (36.55%. 91.04%)	34.02% (20.27%. 50.33%)
β-lactam	Baseline	100%	99.84% (66.17%. 100%)	97.97% (90.2%. 99.75%)
β-lactam	Post-induction	98.81% (70.59%. 100%)	97.11% (43.38%. 100%)	86.69% (73.69%. 94.4%)
β-lactam	Post-consolidation	99.93% (88.63%. 100%)	96.48% (30.63%. 100%)	91.85% (76.42%. 98.08%)
SA1 β-lactamase	Baseline	12.56% (2.68%. 35.79%)	6.87% (0.63%. 31.6%)	10.51% (3.03%. 26.43%)
SA1 β-lactamase	Post-induction	12.55% (4.17%. 28.59%)	15.58% (4.27%. 38.05%)	13.67% (6.41%. 25.16%)
SA1 β-lactamase	Post-consolidation	17.51% (6.77%. 35.39%)	29.48% (8.24%. 62.18%)	21.68% (10.08%. 38.64%)
SA2 β-lactamase	Baseline	100%	100%	97.66% (88.76%. 99.71%)
SA2 β-lactamase	Post-induction	100%	99.95% (10.89%. 100%)	84.04% (70.71%. 92.61%)
SA2 β-lactamase	Post-consolidation	100%	99.73% (6.53%. 100%)	88.38% (73.64%. 96.05%)
MLS	Baseline	100%	99.81% (26.54%. 100%)	95.96% (82.46%. 99.48%)
MLS	Post-induction	99.99% (66.63%. 100%)	98.96% (45.82%. 100%)	92.96% (82.95%. 97.69%)
MLS	Post-consolidation	99.99% (76.01%. 100%)	99.4% (68.62%. 100%)	95.32% (83.45%. 99.14%)
Multidrug	Baseline	13.78% (2.51%. 41.23%)	49.62% (19.59%. 79.88%)	30.07% (15.71%. 48.46%)
Multidrug	Post-induction	10.18% (2.27%. 29.42%)	30.59% (10.51%. 59.41%)	20.67% (11.19%. 33.75%)
Multidrug	Post-consolidation	15.66% (3.09%. 44.07%)	46.11% (16.95%. 77.67%)	28.66% (15.36%. 45.8%)
Sul-Tri	Baseline	85.15% (62.56%. 96.13%)	80.15% (51.84%. 95.03%)	83.02% (68.41%. 92.38%)
Sul-Tri	Post-induction	87.21% (66.48%. 96.76%)	90.75% (68%. 98.55%)	88.53% (74.93%. 95.83%)
Sul-Tri	Post-consolidation	100%	100%	100%
Tetracycline	Baseline	100%	84.67% (57.58%. 96.81%)	93.79% (80.31%. 98.69%)
Tetracycline	Post-induction	96.78% (83.88%. 99.66%)	94.45% (73.66%. 99.47%)	95.92% (86.49%. 99.14%)
Tetracycline	Post-consolidation	95.83% (79.55%. 99.58%)	100%	97.3% (85.14%. 99.75%)
Quinolone	Baseline	7.89% (1.59%. 24.84%)	ND	4.82% (0.99%. 16.01%)
Quinolone	Post-induction	10.59% (3.4%. 25.08%)	6.05% (0.48%. 30.5%)	8.94% (3.07%. 20.64%)
Quinolone	Post-consolidation	15.3% (4.7%. 35.46%)	4.93% (0.33%. 28.02%)	11.55% (3.82%. 26.65%)
TPM	Baseline	3.7% (0.24%. 22.51%)	ND	2.49% (0.21%. 14.37%)
TPM	Post-induction	10.41% (3.17%. 25.43%)	ND	6.88% (2.33%. 16.37%)
TPM	Post-consolidation	15.13% (4.51%. 35.69%)	ND	9.45% (2.59%. 24.76%)
Vancomycin	Baseline	9.22% (0.9%. 38.69%)	13.84% (2.13%. 44.15%)	12.54% (4.34%. 27.95%)
Vancomycin	Post-induction	5.3% (0.33%. 30.24%)	20.63% (5.73%. 47.59%)	12.26% (5.26%. 24.07%)
Vancomycin	Post-consolidation	ND	6.76% (0.31%. 40.18%)	2.63% (0.29%. 13.11%)

Table 6: Within each prophylaxis group and for the whole cohort, generalized linear mixed models (GLMMs) were applied to estimate the prevalence (95% confidence intervals) of each ARG class at each time point during the ALL chemotherapy, with probit as the link function, and subject as random effect. Other covariates included log10-transformed TBR (total bacterial reads) and time points. MLS refers to macrolide, lincosamide and streptogramin antibiotics. Sul-Tri refers to Sulfamethoxazole-Trimethoprim. TPM refers to topisomerase point mutations.

2.7 Estimated mean fold change of abundance of antibiotic resistance gene classes at each time point based on negative binomial GLMMs

ARG	Phase	Levofloxacin	No prophylaxis	All
Aminoglycoside	Induction	5.67 (1.86. 17.27)	18.82 (2.9. 121.99)	10.47 (3.15. 34.75)
β-lactam	Induction	0.65 (0.35. 1.2)	0.65 (0.22. 1.91)	0.63 (0.36. 1.12)
SA1 β-lactamase	Induction	3.87 (0.49. 30.26)	7.33 (0.69. 77.76)	4.37 (0.71. 26.81)
SA2 β-lactamase	Induction	0.62 (0.32. 1.21)	0.66 (0.21. 2.06)	0.61 (0.33. 1.13)
MLS	Induction	1.01 (0.49. 2.09)	2.31 (0.9. 5.92)	1.31 (0.74. 2.33)
Multidrug	Induction	8.08 (5.32. 12.26)	9.32 (3.96. 21.9)	8.55 (5.83. 12.55)
Sul-Tri	Induction	5.1 (2.81. 9.25)	7.42 (3.26. 16.9)	5.88 (3.6. 9.59)
Tetracycline	Induction	0.78 (0.36. 1.7)	1.96 (1. 3.84)	1.04 (0.55. 1.96)
Quinolone	Induction	30.25 (0.16. 5581.56)	NA	18.72 (0.13. 2612.68)
TPM	Induction	37.83 (0.15. 9635.41)	NA	40.3 (0.16. 9850.44)
Vancomycin	Induction	NA	66.16 (0.45. 9754.27)	51.18 (0.96. 2739.33)
Aminoglycoside	Consolidation	0.46 (0.14. 1.57)	0.48 (0.08. 2.7)	0.46 (0.13. 1.59)
β-lactam	Consolidation	1.27 (0.61. 2.62)	0.6 (0.22. 1.65)	1.08 (0.58. 2.01)
SA1 β-lactamase	Consolidation	0.57 (0.08. 3.89)	2.27 (0.27. 18.92)	0.83 (0.17. 4.01)
SA2 β-lactamase	Consolidation	1.32 (0.61. 2.86)	0.53 (0.17. 1.69)	1.1 (0.56. 2.16)
MLS	Consolidation	0.81 (0.45. 1.47)	0.75 (0.23. 2.38)	0.83 (0.48. 1.44)
Multidrug	Consolidation	0.2 (0.02. 1.69)	1.75 (0.32. 9.59)	0.64 (0.13. 3.14)
Sul-Tri	Consolidation	1.7 (0.79. 3.64)	1.84 (0.7. 4.84)	1.75 (0.96. 3.19)
Tetracycline	Consolidation	1.05 (0.69. 1.6)	0.63 (0.33. 1.21)	0.9 (0.63. 1.28)
Quinolone	Consolidation	0.59 (0. 76.69)	NA	2.39 (0.02. 255.8)
TPM	Consolidation	0.59 (0. 103.52)	NA	0.6 (0. 117.21)
Vancomycin	Consolidation	NA	0.01 (0. 0.93)	0.01 (0. 0.27)

Table 7: Within each prophylaxis group and for the whole cohort, GLMMs for the negative binomial family were performed to estimate the fold change in group mean abundances (95% confidence intervals) during each phase of ALL chemotherapy. Specifically, the negative binomial GLMM models with and without prophylaxis group effects included RPKM read counts (rounded to be integers) as response variables, time point as a covariate, and subject as a random factor. Phases of chemotherapy were defined as Induction (between Baseline and Post-Induction samples) and Consolidation (between Post-Induction and Post-Consolidation samples) see Supplemental figure 3.1. MLS refers to macrolide, lincosamide and streptogramin antibiotics. Sul-Tri refers to Sulfamethoxazole-Trimethoprim. TPM refers to topisomerase point mutations.

2.8 Specific quinolone resistance antibiotic resistance genes

Individual	Prophylaxis	ARG	Mechanism	Abundance (RPKM)		
				Baseline	Post Induction	Post Consolid.
A	Levofloxacin	patA	quinolone specific efflux pump	ND	67	ND
B	Levofloxacin	patA	quinolone specific efflux pump	ND	ND	135.45
C	No Prophylaxis	QnrB19	DNA gyrase modifier	434	ND	ND
D	No Prophylaxis	patA	quinolone specific efflux pump	11	277	188

Table 8: Limited number (4 individuals) of quinolone (non-topisomerase point mutation) antibiotic resistance genes were found in this cohort. ND means not detected.

2.9 Association between ARG and microbiome composition

ARG	Phyla	Unadjusted			Adjusted for prophylaxis group		
(log_{10} fold change)	(% relative abundance)	Estimate	SE	P value	Estimate	SE	P value
Multidrug	Proteobacteria	0.03124	0.007677	0.00070	0.03109	0.007699	0.00070
Aminoglycoside	Acinetobacteria	0.005192	0.0074	0.49	0.005037	0.006922	0.48
Quinolone	Proteobacteria	0.02298	0.01237	0.079	0.02357	0.01234	0.072
TPM	Proteobacteria	0.02386	0.01651	0.17	0.02496	0.01563	0.13
Sul-Tri	Proteobacteria	-0.00057	0.01008	0.96	-0.00067	0.01015	0.95
Sul-Tri	Firmicutes	0.007396	0.005976	0.23	0.007159	0.006002	0.25
Beta-lactam	Proteobacteria	-0.00282	0.006256	0.66	-0.00273	0.006251	0.67
SA1	Proteobacteria	0.02258	0.01109	0.056	0.02269	0.01087	0.051
SA2	Bacteroides	0.01472	0.003862	0.0012	0.01529	0.004016	0.0012
Tetracycline	Bacteroides	-0.00302	0.002693	0.28	-0.00205	0.002529	0.43
TetX	Bacteroides	0.01165	0.003425	0.0030	0.01213	0.003646	0.0040
Vancomycin	Bacteroides	-0.00423	0.002329	0.085	-0.00444	0.002345	0.074

Table 9: For microbiome data, changes in relative abundance of bacterial taxa were calculated for each subject. Linear mixed models (LMM) with subject as random effect were then applied to examine the association between changes in selected ARG classes and changes in microbiome composition. Estimate refers to the degree to which log_{10} fold change in ARG reads (RPKM) is associated with % relative abundance change in particular microbiome phylum. Sul-Tri refers to Sulfamethoxazole-Trimethoprim. TPM refers to topisomerase point mutations.

2.10 Association between antibiotic use and antibiotic resistance gene abundance

Antibiotics (days)	ARG (log_{10} fold change)	Estimate	Standard Error	P value
Aminoglycoside	Aminoglycoside	-0.058	0.328	0.86
Total antibiotics	Aminoglycoside	0.004	0.013	0.76
Anti-pseudomonal β-lactam	Aminoglycoside	0.050	0.028	0.080
Aminoglycoside	Multidrug	-0.326	0.308	0.30
Total antibiotics	Multidrug	0.004	0.013	0.75
B. fragilis-active antibiotics	Multidrug	0.068	0.037	0.075
Anti-pseudomonal β-lactam	Multidrug	0.062	0.025	0.021
Quinolone	Quinolone	0.002	0.020	0.93
Total antibiotics	Quinolone	-0.008	0.016	0.63
Anti-pseudomonal β-lactam	Quinolone	0.009	0.034	0.80
Total antibiotics	SA1 β-lactam	0.009	0.010	0.35
B. fragilis-active antibiotics	SA1 β-lactamase	0.020	0.031	0.51
Anti-pseudomonal β-lactam	SA1 β-lactamase	0.014	0.022	0.54
Total antibiotics	SA2 β-lactamase	-0.006	0.013	0.65
B. fragilis-active antibiotics	SA2 β-lactamase	-0.070	0.037	0.069
Anti-pseudomonal β-lactam	SA2 β-lactamase	-0.010	0.028	0.72
Quinolone	TPM	-0.010	0.023	0.67
Total antibiotics	TPM	-0.017	0.017	0.33
Anti-pseudomonal β-lactam	TPM	-0.011	0.038	0.77
Total antibiotics	Vancomycin	0.012	0.011	0.28
Vancomycin	Vancomycin	0.045	0.026	0.093
B. fragilis-active antibiotics	Vancomycin	0.106	0.027	0.00050
Anti-pseudomonal β-lactam	Vancomycin	0.040	0.023	0.086
Total antibiotics	β-lactam	-0.009	0.013	0.49
Vancomycin	β-lactam	-0.017	0.032	0.60
B. fragilis-active antibiotics	β-lactam	-0.066	0.037	0.086
Anti-pseudomonal β-lactam	β-lactam	-0.008	0.028	0.78

Table 10: Linear regression analyses were performed with log10 fold change in ARG during the induction treatment phase as the response variable and prophylaxis group and days of antibiotic as covariates. Estimate refers to the degree to which changes in days on antibiotic altered log_{10} fold change in ARG reads (RPKM). When tested with the interaction between prophylaxis group and days of antibiotic there was no change in the results (data not shown). TPM refers to topisomerase point mutations.

3 Supplemental Figures

3.1 Study time course

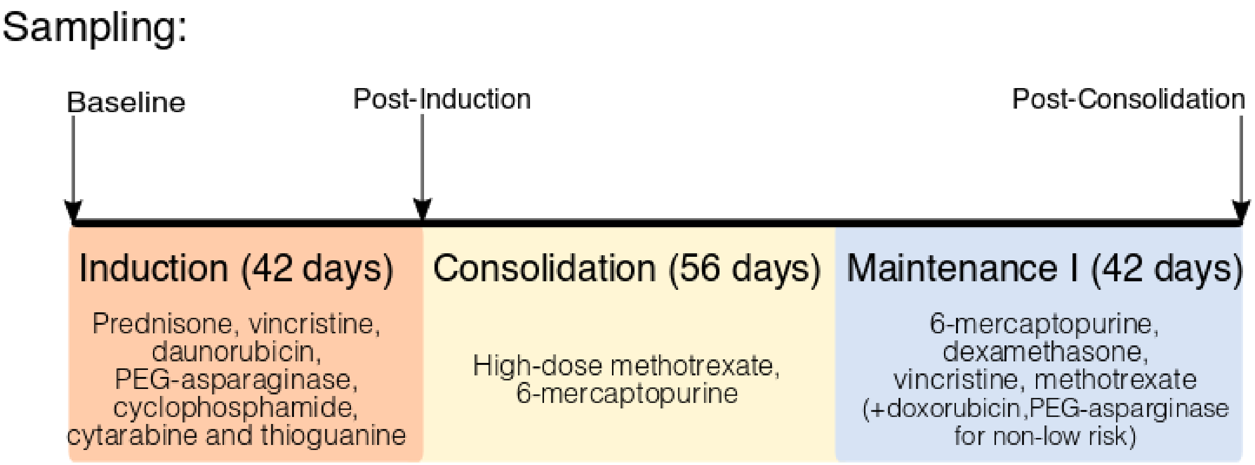

3.2 Flow diagram summarizing eligibility and inclusion

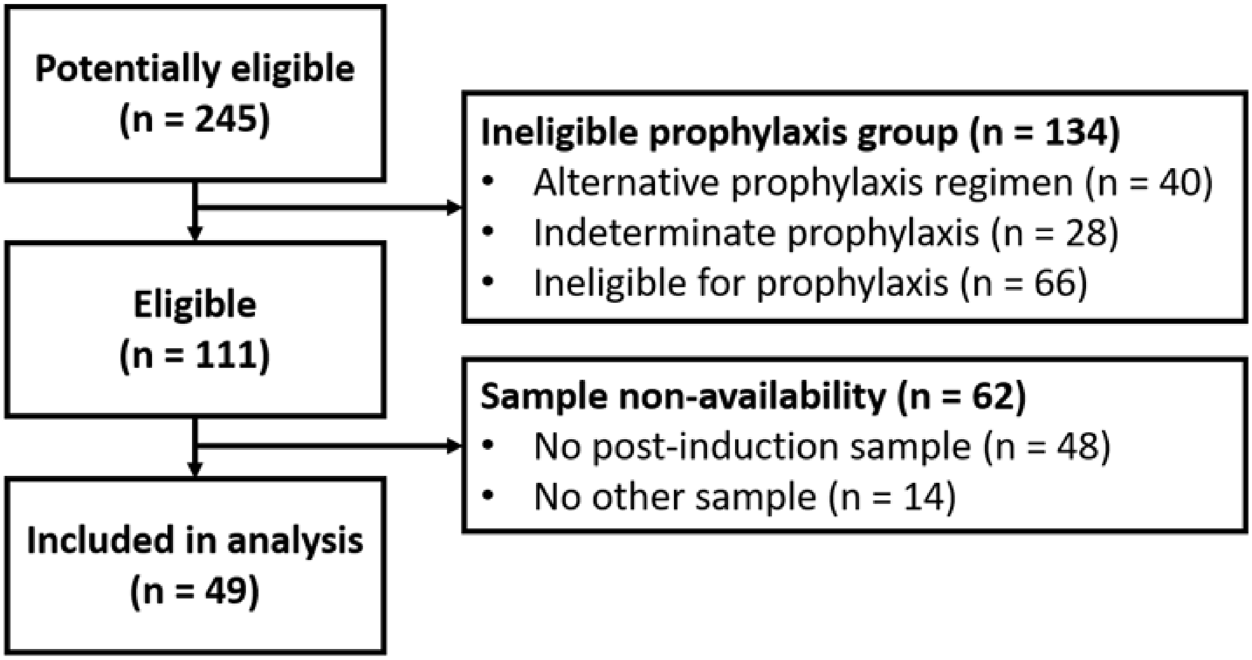

3.3 Serine-β-lactamases class A prevalence and abundance

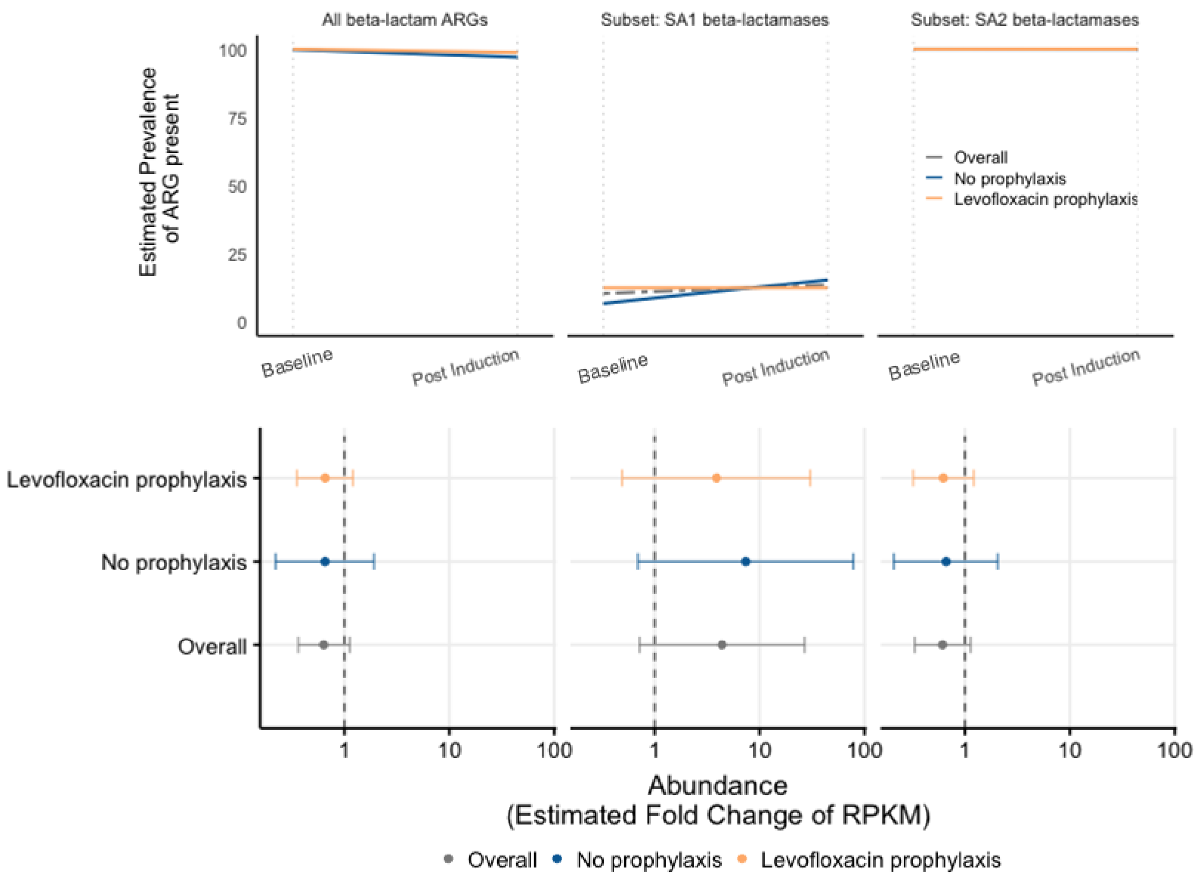

Figure 3: a) The prevalence of all β-lactam ARGs of the subset that belong to the serine-β-lactamases class A subgroup 1 (SA1) and of the subset that belong to the serine-β-lactamases class A subgroup 2 (SA2) estimated from generalized linear mixed models (GLMMs) accounting for the prior time period and sequence depth. b) Distinct trends in the abundance of β-lactam ARGs among subset SA1 and SA2 genes estimated as the fold change in ARG reads per kilobase of ARG per million total bacterial reads from GLMMs regardless of the fluoroquinolone prophylaxis group

4 References

[1] Hakim H, Dallas R, Wolf J, et al. Gut Microbiome Composition Predicts Infection Risk During Chemotherapy in Children With Acute Lymphoblastic Leukemia. *Clin Infect Dis* 2018; 67(4): 541-8.

[2] Jeha S, Pei D, Choi J, et al. Improved CNS Control of Childhood Acute Lymphoblastic Leukemia Without Cranial Irradiation: St Jude Total Therapy Study 16. *J Clin Oncol* 2019; 37(35): 3377-91.

[3] Wolf J, Tang L, Flynn PM, et al. Levofloxacin Prophylaxis During Induction Therapy for Pediatric Acute Lymphoblastic Leukemia. *Clin Infect Dis* 2017; 65(11): 1790-8.

[4] Zaheer R, Noyes N, Ortega Polo R, et al. Impact of sequencing depth on the characterization of the microbiome and resistome. *Sci Rep* 2018; 8(1): 5890.

[5] Wood DE, Salzberg SL. Kraken: ultrafast metagenomic sequence classification using exact alignments. *Genome Biol* 2014; 15(3): R46.

[6] Silveira MC, Azevedo da Silva R, Faria da Mota F, et al. Systematic Identification and Classification of β-Lactamases Based on Sequence Similarity Criteria: β-Lactamase Annotation. *Evolutionary Bioinformatics* 2018; 14: 1176934318797351.
